# Pyroptotic Macrophage Extracellular Vesicles Drive miR‐103a‐3p Mediated Mitochondrial Dysfunction in Granulosa Cells in Diminished Ovarian Reserve

**DOI:** 10.1111/acel.70806

**Published:** 2026-11-05

**Authors:** Wenhan Ju, Xin Xin, Yanli Xiao, Liwen Song, Jinfu Zhang, Shan Xiang

**Affiliations:** ^1^ Department of Gynaecology Guanghua Hospital Affiliated to Shanghai University of Traditional Chinese Medicine Shanghai China; ^2^ Shenzhen Luohu Hospital of Traditional Chinese Medicine, Shenzhen Luohu Hospital Group Shenzhen China; ^3^ Tongren Hospital, Shanghai Jiao Tong University School of Medicine Shanghai China; ^4^ Guanghua Hospital Affiliated to Shanghai University of Traditional Chinese Medicine Shanghai China; ^5^ Reproductive Center of Integrated Medicine Affiliated Hospital of Shandong University of Traditional Chinese Medicine Jinan China; ^6^ The First Clinical College Shandong University of Traditional Chinese Medicine Jinan China

**Keywords:** diminished ovarian reserve, extracellular vesicles, granulosa cells, macrophage, miR‐103a‐3p, mitochondrial dysfunction, pyroptosis

## Abstract

To define the role of pyroptotic macrophage‐derived extracellular vesicles (EVs) in diminished ovarian reserve (DOR) and to elucidate whether EVs‐mediated transfer of miR‐103a‐3p drives mitochondrial dysfunction in granulosa cells. Single‐cell RNA sequencing data from public GEO datasets were analyzed to characterize pyroptosis‐associated features in ovarian macrophages across age groups. EVs were isolated from pyroptotic macrophages and applied to granulosa cells (KGN) to assess mitochondrial function, oxidative stress, and apoptosis. miRNA sequencing was performed to identify EVs‐enriched miRNAs. miR‐103a‐3p expression was further evaluated in follicular fluid EVs from DOR patients and correlated with ovarian reserve markers. Mechanistic studies were conducted using in vitro assays and an in vivo mouse model. Pyroptosis signatures were enriched in ovarian macrophages with aging. EVs from pyroptotic macrophages were enriched in miR‐103a‐3p and induced mitochondrial dysfunction in granulosa cells, characterized by reduced ATP, increased ROS, and enhanced apoptosis; these effects were alleviated by miR‐103a‐3p inhibition. Clinically, miR‐103a‐3p was elevated in follicular fluid EVs from DOR patients and negatively correlated with AMH and AFC. Mechanistically, miR‐103a‐3p targeted *MFN2* and *BCL2L13*, disrupting mitochondrial homeostasis in KGN cells. Restoration of these targets mitigated dysfunction in KGN cells. In vivo, AAV‐mediated inhibition of miR‐103a‐3p improved follicular development and ovarian function. Pyroptotic macrophages may contribute to DOR progression through EVs‐mediated delivery of miR‐103a‐3p, which impairs mitochondrial homeostasis in granulosa cells by targeting *MFN2* and *BCL2L13*.

## Graphical abstract

Pyroptotic macrophages release extracellular vesicles enriched in miR‐103a‐3p. These EVs are taken up by granulosa cells, where miR‐103a‐3p targets MFN2 and BCL2L13, disrupting mitochondrial homeostasis and increasing oxidative stress and apoptosis. This EV‐mediated signaling axis contributes to diminished ovarian reserve and represents a potential therapeutic target.

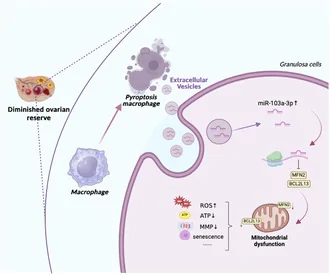

**Abbreviations**
AFC: antral follicle count
AMH: anti‐Müllerian hormone
ART: assisted reproductive technology
ATP: adenosine triphosphate
AUC: area under the curve
BAX: BCL2‐associated X protein
BCL2: B‐cell lymphoma 2
BCL2L13: BCL2‐like 13
BP: biological process
CC: cellular component
DEmRNAs: differentially expressed messenger RNAs
DHE: dihydroethidium
DOR: diminished ovarian reserve
E_2_
: estradiol
EVs: extracellular vesicles
FF: follicular fluid
FSH: follicle‐stimulating hormone
FSHR: follicle‐stimulating hormone receptor
GO: Gene Ontology
GSH: glutathione
IL: interleukin
KEGG: Kyoto Encyclopedia of Genes and Genomes
LDH: lactate dehydrogenase
LH: luteinizing hormone
LHCGR: luteinizing hormone/choriogonadotropin receptor
MAPK: mitogen‐activated protein kinase
MDA: malondialdehyde
MF: molecular function
MFN2: mitofusin 2
miRNA: microRNA
mRNA: messenger RNA
mTOR: mechanistic target of rapamycin
mtROS: mitochondrial reactive oxygen species
NC: negative control
NLRP1: NLR family pyrin domain containing 1
NLRP3: NLR family pyrin domain containing 3
NOR: normal ovarian reserve
NTA: nanoparticle tracking analysis
OS: oxidative stress
PBS: phosphate‐buffered saline
PCR: polymerase chain reaction
Pyr: pyroptotic macrophages
Pyr‐EVs: extracellular vesicles derived from pyroptotic macrophages
qRT‐PCR: quantitative real‐time polymerase chain reaction
RNA‐seq: RNA sequencing
ROC: receiver operating characteristic
ROS: reactive oxygen species
SASP: senescence‐associated secretory phenotype
SA‐β‐gal: senescence‐associated β‐galactosidase
TEM: transmission electron microscopy
TMRE: tetramethylrhodamine ethyl ester
TUNEL: terminal deoxynucleotidyl transferase dUTP nick end labeling
UMAP: Uniform Manifold Approximation and Projection
WT: wild type

## 1 Introduction

Ovarian reserve function is a key indicator of female reproductive potential, primarily reflecting the quantity of available follicles and the quality of oocyte development (Harris et al. 2023). The incidence of diminished ovarian reserve (DOR) among women of reproductive age has been increasing annually, influenced by factors such as delayed childbearing and adverse environmental exposures. Notably, DOR is occurring at progressively younger ages. Clinically, DOR is characterized by elevated basal follicle‐stimulating hormone (FSH) levels (> 10 mIU/mL), reduced antral follicle count (AFC) (< 5–7), and decreased anti‐Müllerian hormone (AMH) concentrations (< 0.5–1.1 ng/mL) (Poseidon Group (Patient‐Oriented Strategies Encompassing IndividualizeD Oocyte Number) et al. 2016; Conforti et al. 2025). Due to the reduced follicular pool and compromised oocyte quality, patients with DOR exhibit a lower natural pregnancy rate. Although assisted reproductive technologies (ART) have provided opportunities for conception in some cases, poor ovarian response, limited oocyte retrieval, and suboptimal embryo quality remain major factors restricting successful pregnancy outcomes (Cedars 2022). Therefore, elucidating the underlying mechanisms of DOR is essential for advancing the prevention and treatment of ovarian dysfunction and safeguarding women's reproductive health.

The normal development of ovarian follicles is closely associated with the functional integrity of granulosa cells. Granulosa cells not only supply metabolic energy to oocytes during follicular growth but also contribute to oocyte development, maturation, and ovulation by secreting hormones and regulating key signaling pathways (Canipari 2000; Martinez et al. 2023). In recent years, non‐coding RNAs—particularly microRNAs (miRNAs)—have garnered considerable attention as critical epigenetic regulators of cellular behavior in ovarian physiology and pathology (Zhang, Xu, et al. 2019; McGinnis et al. 2015). miRNAs modulate the expression of target genes by binding to the 3′‐untranslated region (3′‐UTR) of messenger RNAs (mRNAs), thereby influencing essential cellular processes such as apoptosis, proliferation, and stress responses (Diener et al. 2022; Ferragut Cardoso et al. 2021). Abnormal expression of Dicer, a key enzyme involved in miRNA processing, has been shown to impair follicular development. Notably, *Dicer* knockdown in granulosa cells induces ovarian dysfunction, underscoring the indispensable role of miRNAs in ovarian regulation (Luense et al. 2009). Furthermore, specific miRNAs (miR‐484, miR‐145‐5p, and miR‐6881‐3p) have been reported to play pivotal roles in maintaining ovarian function by modulating granulosa cell apoptosis and proliferation, thereby influencing oocyte quality and embryonic developmental potential (Wang, Yang, et al. 2023; Lu et al. 2023; Ju et al. 2024). Notably, these studies predominantly investigated miRNAs expressed within ovarian cells themselves, whereas the contribution of immune cell‐derived EVs‐associated miRNAs to ovarian aging remains poorly understood.

Oxidative stress (OS) is widely recognized as a key pathogenic factor contributing to the onset and progression of ovarian failure, particularly DOR (Ammar et al. 2024). Under physiological conditions, reactive oxygen species (ROS) participate in cellular signaling regulation; however, when ROS production exceeds the neutralizing capacity of the cellular antioxidant system, oxidative damage occurs (D'Autréaux and Toledano 2007). Excessive ROS accumulation can directly disrupt mitochondrial membrane potential and impair respiratory chain function, resulting in altered mitochondrial membrane permeability. This, in turn, induces mitochondria‐mediated apoptosis of granulosa cells, accelerates follicular atresia and depletion, and consequently diminishes ovarian reserve capacity (Wang et al. 2021). Notably, miRNAs have emerged as crucial regulators in the cellular response to oxidative stress, attracting increasing attention in reproductive biology research. A growing body of evidence indicates that miRNAs modulate ovarian cell injury by targeting apoptosis‐related genes, antioxidant defense components, and mitochondrial function‐associated proteins (Wang, Yang, et al. 2023; Zhu et al. 2024; Zhang et al. 2017). Thus, miRNA‐mediated epigenetic regulation represents a promising avenue for elucidating the molecular interplay between oxidative stress and ovarian dysfunction.

Recent studies have demonstrated that the ovary is not only a reproductive organ but also an important immunoregulatory microenvironment. Within this niche, macrophages play a central role in maintaining tissue homeostasis, clearing apoptotic cells, and modulating inflammatory responses. With advancing age, the ovarian immune microenvironment gradually becomes dysregulated, characterized by enhanced pro‐inflammatory activity and altered immune cell function. Previous studies have largely focused on ovarian aging, primarily involving women of advanced age. Notably, the biological features of ovarian aging substantially overlap with those of age‐related DOR, rendering these findings highly relevant for understanding DOR in older individuals. Single‐cell transcriptomic analyses have revealed that pyroptosis‐related pathways are significantly activated in ovarian macrophages during aging, driving a shift in the local microenvironment from immune homeostasis toward a pro‐inflammatory state (Zhou et al. 2024; Shen et al. 2024). In addition, macrophage dysfunction and the accumulation of inflammatory mediators have been implicated in the decline of ovarian function. Modulation of macrophage differentiation and inflammatory polarization—such as via the SIRT5‐RAC2 axis—has been shown to markedly influence ovarian inflammation, follicular preservation, and functional maintenance, further underscoring the critical role of immune cells in age‐related DOR (TanTai et al. 2026). Moreover, macrophages can secrete extracellular vesicles (EVs) to deliver bioactive molecules, including miRNAs, to target cells, thereby regulating mitochondrial function, proliferation, and apoptosis (Huang et al. 2021; Nguyen et al. 2018). This EVs‐mediated intercellular communication provides a novel perspective for elucidating the mechanisms underlying DOR.

Our previous study demonstrated that the expression of miR‐103a‐3p is significantly upregulated in follicular fluid granulosa cells of women with DOR (Liu et al. 2023). Existing study suggests that high expression of miR‐103a‐3p in follicular fluid is associated with poorer embryo quality in IVF‐ET treatments (Zhang et al. 2021). In Parkinson's disease models, miR‐103a‐3p exacerbates neuronal damage by inhibiting the Parkin/Ambra1 signaling pathway, thereby impairing mitophagy (Zhou et al. 2020). Similarly, in a cerebral ischemia–reperfusion model, miR‐103a‐3p regulates inflammation, oxidative stress, and apoptosis by targeting the *HMGB1* mRNA, consequently mitigating tissue injury (Li et al. 2020). However, despite growing evidence supporting the involvement of miRNAs in ovarian aging and DOR, previous studies have primarily focused on miRNAs expressed within granulosa cells (GCs) or oocytes. Whether pathogenic miRNAs can originate from immune cells and be transferred to ovarian somatic cells through extracellular vesicle‐mediated communication remains largely unknown. In particular, the contribution of pyroptotic macrophages to miRNA‐driven ovarian dysfunction has not been investigated.

Given our previous observation that miR‐103a‐3p is upregulated in granulosa cells from women with DOR, we hypothesized that this miRNA may not only function as an intracellular regulator but may also serve as an extracellular signaling molecule derived from inflammatory macrophages. Therefore, the present study integrates public single‐cell RNA sequencing analyses with in vitro and in vivo experiments to determine whether pyroptotic macrophage‐derived EVs deliver miR‐103a‐3p to granulosa cells and to elucidate the downstream mechanisms by which this immune‐to‐ovarian signaling axis contributes to mitochondrial dysfunction and DOR progression.

## 2 Methods

### 2.1 Preprocessing of Single‐Cell RNA‐Seq Data and Macrophage Identification

Raw gene expression matrices from four 10× Genomics samples (GSE267315) were downloaded from GEO and analyzed using Seurat (v4). Cells with < 200 or > 6000 detected genes, > 10% mitochondrial genes, or genes expressed in < 3 cells were excluded. Data were log‐normalized, and 3000 highly variable genes were identified. After scaling and regression of mitochondrial gene content, macrophages were annotated based on canonical markers (*C1QA*, *C1QB*, *C1QC*, *CD68*, *MS4A7*, *APOE*, *MRC1*, *LYVE1*). Pyroptotic activity was quantified using a curated gene set (*CASP1*, *GSDMD, IL1B*, *NLRP3*, *PYCARD*, *IL18*, *NLRP1*), and a pyroptosis score was calculated with the Seurat AddModuleScore function to assess relative activity across cell populations.

### 2.2 Clinical Data Analysis

#### 2.2.1 Patients

This study was approved by the Ethics Committee of Reproductive Medicine of the affiliated hospital of Shandong University of Traditional Chinese Medicine, and written informed consent was obtained from all participants. The study was registered in the China Clinical Trial Registry (registration number: ChiCTR1800019798, https://www.chictr.org.cn/index.html). Thirty patients aged ≥ 38 years and diagnosed with DOR, who underwent in vitro fertilization or intracytoplasmic sperm injection (IVF/ICSI) at the Center for Reproduction and Genetics, Affiliated Hospital of Shandong University of Traditional Chinese Medicine, between January 1, 2019, and May 31, 2019, were recruited. The diagnosis of DOR was based on the Bologna criteria (Ferraretti et al. 2011) (AFC < 5–7 or AMH < 0.5–1.1 μg/L). Thirty patients with NOR who underwent ICSI due to male factor infertility were included as controls. The baseline clinical characteristics of all participants are presented in Supplementary Table 1. Patients with comorbidities potentially affecting oocyte quality, such as endometriosis, hyperthyroidism, or hyperprolactinemia, were excluded. Individuals with severe systemic diseases, hematologic disorders, ovarian tumors, or other malignancies were also excluded from participation.

#### 2.2.2 Treatment and Embryo Evaluation

All patients underwent controlled ovarian stimulation using a gonadotropin‐releasing hormone (GnRH) antagonist protocol. On Day 2 of the menstrual cycle, baseline reproductive hormone levels were measured, and transvaginal ultrasonography was performed. Ovarian stimulation was initiated according to patient‐specific parameters, including age, body mass index (BMI), antral follicle count (AFC), and basal follicle size. Patients received daily subcutaneous injections of 225–300 IU recombinant follicle‐stimulating hormone (r‐FSH; Puregon, 600 IU, Merck Sharp & Dohme). Follicular development was monitored every 2–4 days by transvaginal ultrasound and serum hormone assessment, and r‐FSH dosages were adjusted accordingly. When the leading follicle reached 12–14 mm in diameter, a daily subcutaneous injection of ganirelix acetate (0.25 mg; Orgalutran, Merck Sharp & Dohme) was administered until the ovulation trigger day. When the dominant follicle reached 18 mm, ovulation was triggered with a single injection of recombinant human chorionic gonadotropin (rhCG; Ovidrel, 250 μg) at 8:30 p.m. Oocyte retrieval was performed 36 h later under transvaginal ultrasound guidance, and follicular fluid was collected for analysis.

In this study, embryo quality was assessed based on standard morphological criteria. Normal fertilization was defined as the presence of two pronuclei (2PN) and two polar bodies at 16–18 h post‐insemination(Alpha Scientists in Reproductive Medicine and ESHRE Special Interest Group of Embryology 2011). Cleavage‐stage embryos were evaluated according to cell number, blastomere symmetry, and degree of fragmentation. Usable embryos were typically defined as those with ≥ 6 cells on Day 3 and ≤ 25% fragmentation, without multinucleation. Blastocyst quality was assessed using the Gardner grading system, which evaluates three parameters: degree of blastocoel expansion (1–6), inner cell mass (ICM), and trophectoderm (TE) (Gardner et al. 2000). Blastocysts with grades AA, AB, or BA were classified as high‐quality embryos (Zou et al. 2023).

#### 2.2.3 Follicular Fluid Granulosa Cell Extraction

Follicular fluid samples were collected and centrifuged at 3000 rpm for 10 min. The cell pellet was resuspended in 4 mL of phosphate‐buffered saline (PBS; 1×) to obtain a uniform cell suspension. The suspension was then carefully layered over 2.5 mL of human lymphocyte separation medium and centrifuged at 2000 rpm for 10 min. The white, cloudy interphase layer containing granulosa cells was aspirated into a 1.5 mL centrifuge tube, washed once with 1× PBS, and centrifuged at 2500 rpm for 10 min. After discarding the supernatant, the resulting cells were collected as the granulosa cell fraction from the follicular fluid.

#### 2.2.4 Isolation and Characterization of Follicular Fluid‐Derived Extracellular Vesicles

Follicular fluid was first centrifuged at 3000 r/min for 10 min to remove cellular debris, and the supernatant was collected. The supernatant was then centrifuged at 10,000 × g for 30 min, and the resulting supernatant was filtered through a 0.22 μm filter (Millex‐GP, Merck Millipore Ltd.). The filtered supernatant was subjected to ultracentrifugation at 100,000 × g for 70 min (Thermo Scientific Sorvall WX+), and the pellet was collected. The pellet was resuspended in PBS and centrifuged again at 100,000 × g for 90 min. Finally, the pellet was resuspended in 200 μL PBS to obtain the EVs. The morphology of the EVs was observed using transmission electron microscopy (HT7700, HITACHI Ltd., Tokyo, Japan). Nanoparticle tracking analysis (NTA) was performed to determine particle size and concentration. Western blot analysis confirmed the presence of EV markers CD9, CD63, and CD81, and the absence of the negative marker Calnexin.

### 2.3 Animals and Experimental Design

#### 2.3.1 Experimental Grouping

All animal procedures and experimental protocols were approved by the Laboratory Animal Management and Use Committee of Tongren Hospital, Shanghai Jiao Tong University School of Medicine (Approval No. AF/SQ‐08/04.0). Three‐week‐old female C57BL/6J mice were obtained from Jiangsu Jicui Pharmachem Biotechnology Co. Ltd. The animals were housed in a temperature‐controlled environment (23°C ± 2°C) under a 12‐h light/dark cycle, with ad libitum access to food and water. After a two‐week acclimatization period, forty female C57BL/6J mice were randomly assigned to four groups (*n* = 10 per group): (1) control group; (2) OS group; (3) OS + AAV‐vector group; and (4) OS + AAV‐inhibitor group.

#### 2.3.2 Animal Model Construction and Drug Administration

The administration method and dosage were determined based on previously published studies (Wang, Yang, et al. 2023; Kano et al. 2017). Flag‐labeled adeno‐associated virus 9 (AAV9) was synthesized by Shandong Gene&Bio Co.Ltd. (Shandong, China). At 5 weeks of age, mice received intraperitoneal injections of AAV9 at a concentration of 1.17 × 10^13^ VG/mL containing either miR‐103a‐3p‐specific short hairpin RNA (miR‐103a‐3p inhibitor) to establish ovary‐specific miR‐103a‐3p knockdown models. Mice injected with Flag‐tagged AAV9 (AAV vector) served as controls.

To establish the OS model, mice received intraperitoneal injections of phosphate‐buffered saline (PBS)‐diluted 3‐nitropropionic acid (3‐NP; Sigma, USA) at a dose of 20 mg/kg once daily for 2 weeks starting at 8 weeks of age. Control mice were injected intraperitoneally with an equal volume of PBS. Vaginal smears were collected daily at 9:00 a.m. during the 3‐NP treatment period to monitor the estrous cycle.

At the end of the experiment, C57BL/6J mice were euthanized following a 4‐h fasting period. Anesthesia was induced by intraperitoneal injection of sodium pentobarbital at a dose of 150 mg/kg body weight. Blood samples were collected via the suborbital vein, after which the mice underwent thoracotomy for bilateral ovary excision. Surrounding adipose tissue was removed, and the ovaries were divided as required for subsequent analyses or fixation. All procedures were conducted in strict accordance with institutional animal ethics guidelines, ensuring rapid operation and minimizing animal distress. The sample size (*n* = 10 per group) was determined based on previous studies and preliminary experiments, which indicated that this number was sufficient to detect statistically significant differences (Kanatsu‐Shinohara et al. 2022).

### 2.4 Cell Culture and Intervention

KGN cells were obtained from iCell Bioscience Inc. (Shanghai, China) and cultured in Dulbecco's Modified Eagle Medium/Nutrient Mixture F‐12 (DMEM/F12; Gibco) supplemented with 10% fetal bovine serum (FBS; Gibco) and 1% penicillin–streptomycin (Gibco) at 37°C in a humidified incubator with 5% CO_2_. Cells were seeded in six‐well plates at a density of 3 × 10^5^ cells per well. When the cultures reached approximately 60% confluence, KGN cells were transfected with miR‐103a‐3p mimics, miR‐103a‐3p inhibitor, or miRNA negative control (miRNA NC) using Lipofectamine 3000 (Invitrogen, USA) following the manufacturer's instructions. After 48 h of transfection, the cells were collected for subsequent analyses. To induce oxidative stress, cells were treated with 10 mM 3‐NP diluted in PBS for 24 h.

THP‐1 cells were cultured in RPMI‐1640 medium supplemented with 10% fetal bovine serum (FBS) and 1% penicillin/streptomycin at 37°C in a humidified atmosphere containing 5% CO_2_. THP‐1 cells were treated with 100 ng/mL phorbol 12‐myristate 13‐acetate (PMA) to induce differentiation into macrophage‐like cells, characterized by adherent growth and typical morphological changes (Zhou et al. 2024). Subsequently, cells were further stimulated with tumor necrosis factor‐α (TNF‐α) in combination with interferon‐γ (IFN‐γ) to induce a pyroptotic macrophage phenotype.

THP‐1 cells were cultured in RPMI‐1640 medium supplemented with 10% fetal bovine serum (FBS) and 1% penicillin/streptomycin at 37°C in a humidified atmosphere containing 5% CO_2_. THP‐1 cells were treated with 100 ng/mL PMA for 24 h to induce differentiation into macrophage‐like cells, characterized by adherent growth and typical morphological changes (Zhou et al. 2024). Subsequently, differentiated macrophages were co‐treated with TNF‐α (50 ng/mL) and IFN‐γ (100 ng/mL) for 24 h to induce pyroptosis. Successful establishment of the pyroptotic macrophage model was confirmed by increased GSDMD cleavage, elevated LDH release, and enhanced secretion of IL‐1β and IL‐18.

### 2.5 miRNA Sequencing and Differential Expression Analysis

EVs were isolated from culture supernatants by ultracentrifugation, characterized by TEM, NTA, and Western blotting for CD9, CD63, and CD81. Total RNA was extracted using the miRNeasy Mini Kit (Qiagen, Germany) and assessed by NanoDrop and Bioanalyzer. Small RNA libraries were prepared with the TruSeq Small RNA Library Prep Kit (Illumina, USA) and sequenced on an Illumina NovaSeq 6000 (single‐end 50 bp). Reads were trimmed, filtered for quality, and mapped to the reference genome using Bowtie; rRNA, tRNA, snRNA, and snoRNA were removed via Rfam. Known and novel miRNAs were identified with miRBase and miRDeep2, and expression was normalized to TPM. Differential expression was analyzed with DESeq2, using |log2(fold change)| ≥ 1 and FDR < 0.05 as significance thresholds. Three biological replicates were included per group.

### 2.6 Transcriptome Sequencing

Total RNA from KGN cells was extracted using TRIzol (Invitrogen, USA) and assessed for concentration, purity, and integrity (NanoDrop 2000 and Agilent 2100 Bioanalyzer). Samples with RNA ≥ 100 ng/μL, OD_260/280_ 1.8–2.2, and RIN ≥ 7.0 were used for library preparation. mRNA libraries were constructed with the NEBNext Ultra II RNA Library Prep Kit (NEB, USA) following standard procedures, including RNA fragmentation, cDNA synthesis, end repair, adapter ligation, and PCR amplification. Library quality was checked using Qubit and Bioanalyzer, and qualified libraries were sequenced as paired‐end 150 bp reads on a DNBSEQ‐T7 platform. Raw reads were cleaned with fastp, aligned to the human genome (GRCh38) using HISAT2, and gene counts were obtained with featureCounts or HTSeq. Differential expression analysis was performed with DESeq2, with |fold change| ≥ 2 and Padj < 0.01 considered significant.

In addition, functional enrichment analyses were performed using the DAVID 6.8 database (https://david.ncifcrf.gov/) (Dennis et al. 2003). Gene Ontology (GO) enrichment analysis and Kyoto Encyclopedia of Genes and Genomes (KEGG) pathway enrichment analysis were conducted to identify the biological significance of the differentially expressed genes. The GO analysis included three categories: biological process (BP), molecular function (MF), and cellular component (CC). The organism was set to *Homo sapiens* , and terms with *p* < 0.05 were considered significantly enriched.

### 2.7 miR‐103a‐3p Target Gene Prediction

The potential downstream target genes of miR‐103a‐3p were predicted using three online databases: DIANA (https://diana.imis.athena‐innovation.gr/DianaTools/index.php) (Kavakiotis et al. 2022), TargetScan (https://www.targetscan.org) (McGeary et al. 2019), and miRDB (https://www.mirdb.org/) (Chen and Wang 2020). The intersecting target genes identified across these databases were visualized using a Venn diagram analysis.

### 2.8 Dual Luciferase Reporter

The miR‐103a‐3p mimics, miRNA NC, pmirGLO‐*MFN2* wild‐type (WT) and mutant (MT) vectors, and pmirGLO‐*BCL2L13* WT and mutant MT vectors were synthesized by Shandong Gene&Bio Co.Ltd. (Shandong, China). The sequences used were as follows: miR‐103a‐3p mimics: 3′‐*AGCAGCAUUGUACAGGGCUAUGA*‐5′, miRNA NC: 3′‐*UCUACUCUUUUCUAGGAGGUUGUGA*‐5′. KGN cells in the logarithmic growth phase were collected, resuspended, and seeded into 24‐well plates. When cell confluence reached approximately 70%–80%, the cells were co‐transfected with the appropriate plasmid vectors using Lipofectamine 3000 (Invitrogen, USA) according to the manufacturer's instructions. After 48 h of transfection, firefly and Renilla luciferase activities were measured using the Dual‐Luciferase Reporter Assay Kit (BioVision, USA). Relative luciferase activity was calculated as the ratio of firefly to Renilla luciferase luminescence.

### 2.9 Oxidative Stress and Mitochondrial Function Assays

Oxidative stress and mitochondrial function were evaluated using multiple biochemical and imaging indicators. Total intracellular reactive oxygen species (ROS) levels were measured with a dihydroethidium (DHE) fluorescent probe (C1300, Applygen, China), and mitochondrial ROS (mtROS) were detected using a MitoSOX Red probe (S0061S, Beyotime, China). Fluorescence signals were quantified by flow cytometry (CANTO II, BD Biosciences, USA). Lipid peroxidation was assessed using a malondialdehyde (MDA) thiobarbituric acid (TBA) assay (G4300/G4302, Servicebio, China). The intracellular antioxidant capacity was evaluated by measuring reduced glutathione (GSH) levels using a commercial assay kit (G4305, Servicebio, China). Cellular energy metabolism was determined by quantifying adenosine triphosphate (ATP) content using an ATP assay kit (S0027, Beyotime, China).

Mitochondrial membrane potential (Δψm) was assessed using a tetramethylrhodamine ethyl ester (TMRE) fluorescent probe (C2001S, Beyotime, China). Fluorescence images were captured with an inverted fluorescence microscope, and quantitative analysis was performed by flow cytometry to evaluate mitochondrial function. In addition, mitochondrial morphology and ultrastructure were examined by transmission electron microscopy (TEM) to assess structural alterations in mitochondria (model HT7700, HITACHI Ltd., Tokyo, Japan).

### 2.10 Apoptosis Detection

Apoptosis was assessed by Annexin V‐FITC/PI staining. Cells were digested, washed with PBS, and resuspended in binding buffer, followed by incubation with Annexin V‐FITC for 15 min at room temperature in the dark and PI for 5 min at 4°C. Samples were analyzed by flow cytometry (Canto II, BD Biosciences).

Apoptosis in ovarian tissues was detected using the Click‐iT Plus TUNEL Alexa Fluor 488 Kit (Thermo Fisher Scientific). Paraffin sections were dewaxed, rehydrated, permeabilized with 0.2% Triton X‐100, and incubated with TUNEL reaction mixture at 37°C for 60 min in the dark. Nuclei were counterstained with DAPI, and signals were visualized using a NanoZoomer S60 system. Apoptotic index was quantified based on TUNEL‐positive cells.

### 2.11 Detection of Cellular Senescence

Cellular senescence was evaluated in accordance with the Minimum Information for Cellular Senescence Experimentation in vivo (MICSE) guidelines (Ogrodnik et al. 2024). In KGN cells, senescent cells were identified using a β‐galactosidase (SA‐β‐gal) staining kit (C0602, Beyotime, China). The mRNA expression levels of senescence‐associated secretory phenotype (SASP) factors *IL6* and *IL8* were determined by qRT‐PCR, while the protein expression levels of p16 and p21 were assessed by Western blot.

For mouse ovarian tissues, the mRNA levels of *IL6* and *CXCL1* (the murine functional homolog of *IL8*) were quantified by qRT‐PCR, and the protein expression of p16 and p21 was evaluated by Western blot analysis. In addition, the assessment of oxidative stress was performed as an auxiliary indicator of cellular senescence in this study.

### 2.12 Measurement of Hormone Levels

For KGN cells, testosterone (Sigma, T1500) was added to the culture medium at a final concentration of 10 nM as the substrate for estradiol synthesis (Shao et al. 2022). After 24 h of incubation, the concentration of 17β‐estradiol (17β‐E_2_) in the culture supernatant was determined using a human 17β‐E_2_ ELISA kit (BYabscience, BY‐EH110011).

Serum levels of luteinizing hormone (LH; BYabscience, BY‐EM221468), anti‐Müllerian hormone (AMH; BYabscience, BY‐EM220514), follicle‐stimulating hormone (FSH; BYabscience, BY‐WJZF1604), and estradiol (E_2_; BYabscience, BY‐WJZF0048) were measured using commercial ELISA kits, following the manufacturer's protocols.

### 2.13 HE Staining

Paraffin‐embedded ovarian tissues fixed in 4% paraformaldehyde were sectioned and subsequently dewaxed and rehydrated. Sections were stained with hematoxylin for 3–5 min, rinsed under running water, and differentiated with 0.5% hydrochloric acid‐alcohol until the background was clear. The sections were then counterstained with eosin for 1–3 min, followed by rinsing, dehydration, clearing, and sealing. Tissue morphology was observed and imaged using a NanoZoomer S60 digital slide scanner (C13210‐01, Hamamatsu Photonics K.K., Japan).

### 2.14 Quantitative Real‐Time Polymerase Chain Reaction (qRT‐PCR)

Primer sequences used in this study are listed in Supplementary Table 2. Total RNA was extracted using TRIzol reagent (G3013, Servicebio, China), and reverse transcription was carried out using the PrimeScript RT Reagent Kit (RR037, Takara, Japan). Quantitative real‐time PCR was performed using PowerUp SYBR Green Master Mix (A25743, Thermo Fisher Scientific, USA) following the manufacturer's protocol. The relative expression levels of miRNAs were normalized to *U6*, and those of mRNAs were normalized to *β‐actin*. All experiments were conducted in triplicate. Relative gene expression was calculated using the 2^−ΔΔCt^ method.

### 2.15 Western Blot

Total protein was extracted from cells or tissues using RIPA lysis buffer (P0013B, Beyotime, China). Protein concentrations were determined using the Omni‐Easy Ready‐to‐Use BCA Protein Quantification Kit (ZJ102, Epizyme Biotech, China). Equal amounts of protein (30 μg per sample) were separated by SDS‐PAGE and subsequently transferred onto PVDF membranes. The membranes were blocked with 5% skim milk in TBST for 1 h at room temperature with gentle agitation. The membranes were then incubated overnight at 4°C with the following primary antibodies: MFN2 (1:5000; 12186‐1‐AP, Proteintech), BCL2L13 (1:1000; 16612‐1‐AP, Proteintech), BCL2 (1:5000; 68,103‐1‐Ig, Proteintech), BAX (1:20000; 50599‐2‐Ig, Proteintech), CYP11A1 (1:1000; 13363‐1‐AP, Proteintech), FSHR (1:500; 22665‐1‐AP, Proteintech), LHCGR (1:500; 19968‐1‐AP, Proteintech), p21 (1:1000; 10355‐1‐AP/28248‐1‐AP, Proteintech), and p16 (1:1000; 10883‐1‐AP/28416‐1‐AP, Proteintech). After washing with TBST, the membranes were incubated for 1 h at room temperature with the appropriate HRP‐conjugated secondary antibody (Goat Anti‐Rabbit, 1:5000, SA00001‐2; or Goat Anti‐Mouse, 1:5000, SA00001‐1; Proteintech). Protein bands were visualized using an enhanced chemiluminescence (ECL) detection reagent, and band intensities were quantified using ImageJ software. All primary and secondary antibodies used in Western blot experiments were from the same lot across replicates to ensure experimental consistency.

### 2.16 Statistical Analysis

Experimental data conforming to a normal distribution are presented as mean ± standard deviation (SD). Statistical significance was assessed using the Student's *t*‐test or one‐way ANOVA, followed by Tukey's multiple comparison test, performed with GraphPad Prism software. Non‐normally distributed data are presented as the median (25th–75th percentile), and between‐group comparisons were conducted using the Mann–Whitney test. A *p* < 0.05 was considered statistically significant.

## 3 Results

### 3.1 Single‐Cell Analysis Reveals Enhanced Pyroptosis in Ovarian Macrophages During Aging and Identifies miR‐103a‐3p‐Enriched Extracellular Vesicles

Single‐cell RNA sequencing analysis revealed that, compared with women of reproductive age (age 32/34), macrophages in ovarian tissue from advanced‐age women (age 42) exhibited significantly enhanced pyroptosis‐related features. In particular, IL18, NLRP3, and NLRP1 were differentially expressed (Figure 1A–F; S1A–D). In vitro, THP‐1 cells were induced to differentiate into macrophage‐like cells (M0), followed by stimulation to generate a pyroptotic macrophage phenotype (Pyr). Pyroptosis‐associated signaling was markedly upregulated, as evidenced by increased expression of cleaved caspase‐1 (p20), elevated levels of cleaved GSDMD, and increased secretion of IL‐1β, IL‐18, and LDH in the culture supernatant. These results confirmed the successful establishment of the pyroptotic macrophage model (Figure 1G–J; Figure S1E–F).

**FIGURE 1:**
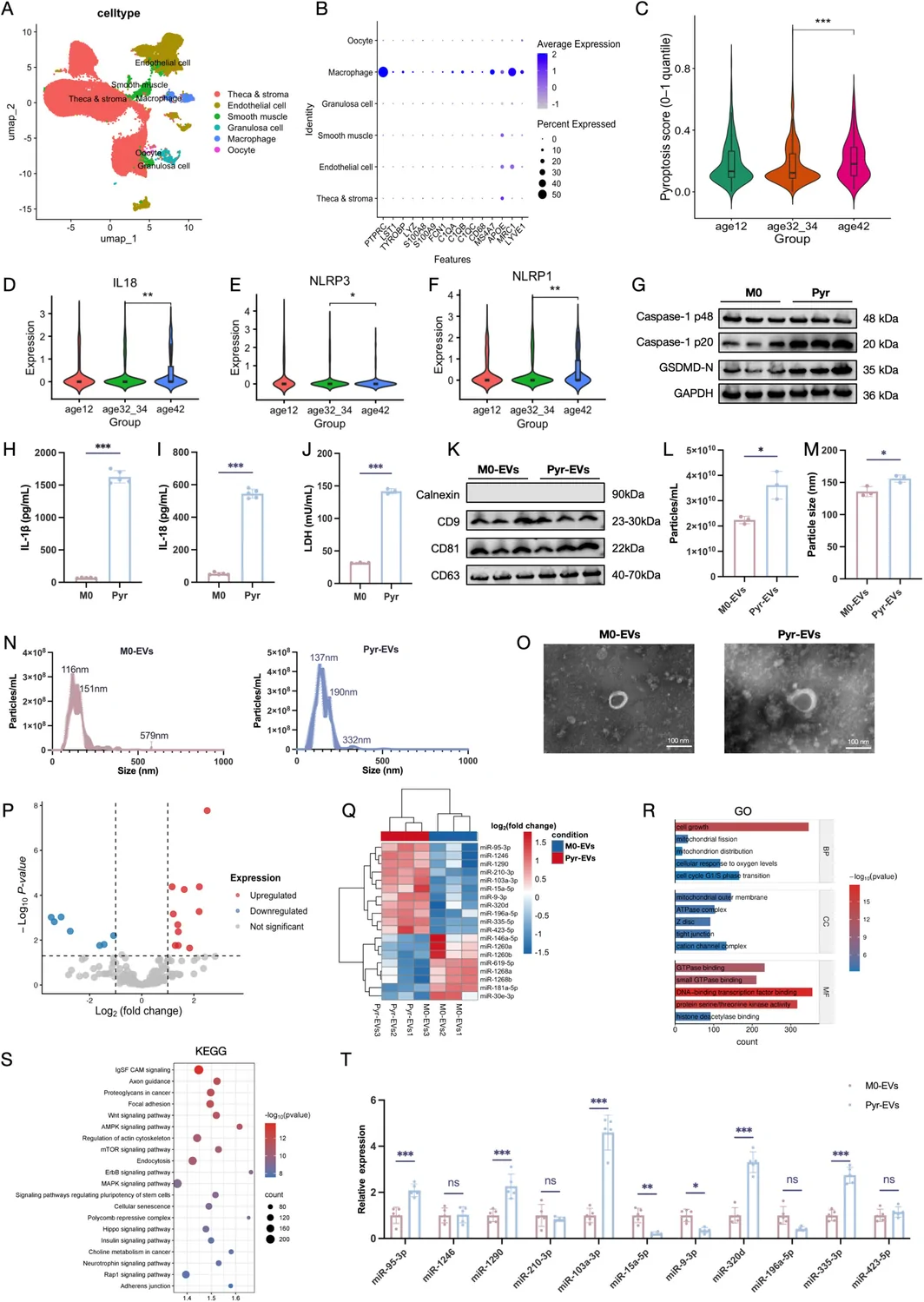
Single‐cell analysis reveals enhanced pyroptosis of macrophages in aged ovaries and identifies miR‐103a‐3p‐enriched extracellular vesicles. (A) UMAP plot showing six major cell types from the single‐cell RNA‐seq dataset GSE267315. (B) Dot plot displaying the differential expression of macrophage marker genes across different cell types. (C) Pyroptosis marker gene expression scores in macrophages from different groups. (D–F) Expression levels of *IL‐18*, *NLRP3*, and *NLRP1* in macrophages across groups. (G) Western blot analysis of Caspase‐1 and GSDMD‐N protein expression (*n* = 3). (H–J) Levels of IL‐1β, IL‐18, and LDH in cell supernatants (*n* = 6). (K) Western blot detection of EVs markers CD9, CD81, and CD63, with Calnexin as a negative control (*n* = 3). (L–N) NTA analysis of EVs particle size, comparing diameter and concentration (*n* = 3). (O) TEM images showing EVs morphology. (P) Volcano plot of miRNA sequencing data (*n* = 3). (Q) Heatmap of differentially expressed miRNAs (*n* = 3). (R) GO enrichment analysis of predicted downstream target genes of miRNAs (*n* = 3). (S) KEGG pathway enrichment analysis of predicted miRNA target genes (*n* = 3). (T) qPCR validation of upregulated miRNAs carried by Pyr‐EVs (*n* = 5). Data are presented as mean ± SD. Replication numbers are indicated in the corresponding panels. Statistical significance was determined using an unpaired two‐tailed Student's *t*‐test. ^ns^ *p* > 0.05, *p < 0.05, **p < 0.01, ***p < 0.001.

EVs were isolated from the culture supernatant. Western blot analysis confirmed the presence of EVs markers. NTA showed that the particle size was mainly distributed within the range of 50–150 nm, and TEM revealed typical round vesicular structures (Figure 1K–O). Compared with EVs derived from M0 macrophages (M0‐EVs), Pyr‐EVs exhibited significantly increased particle size and concentration (Figure 1L,M).

miRNA sequencing identified 19 differentially expressed miRNAs in Pyr‐EVs compared with M0‐EVs, including 11 upregulated and 8 downregulated miRNAs (Figure 1P,Q), based on the criteria of |log2(fold change)| ≥ 1 and adjusted *p‐value* (FDR) < 0.05. GO enrichment analysis suggested that the predicted target genes of these miRNAs are primarily involved in mitochondrial function and energy metabolism, cell growth and proliferation, oxidative stress responses, signal transduction, and epigenetic regulation (Figure 1R). KEGG pathway analysis further indicated enrichment in pathways related to cell proliferation and senescence (e.g., cellular senescence, Wnt, MAPK, Hippo), energy metabolism and nutrient sensing (e.g., AMPK, mTOR, insulin signaling), cytoskeleton organization and adhesion (e.g., focal adhesion, actin cytoskeleton), as well as stress responses and intercellular communication (Figure 1S). qRT‐PCR validation demonstrated that miR‐103a‐3p was the most highly expressed miRNA enriched in Pyr‐EVs (Figure 1T).

### 3.2 Pyr‐EVs Induce Mitochondrial Dysfunction and Apoptosis in KGN Cells via miR‐103a‐3p and Its Downstream Targets

Further experiments revealed that both M0‐EVs and Pyr‐EVs could be internalized by ovarian granulosa cells (KGN) (Figure 2A). Treatment with Pyr‐EVs led to elevated miR‐103a‐3p levels, which were reversed by miR‐103a‐3p inhibitor treatment (Figure S2A). Compared with M0‐EVs, Pyr‐EVs‐treated KGN cells exhibited increased apoptosis (Figure 2B,C), impaired mitochondrial function (evidenced by elevated ROS and MDA levels, and decreased mitochondrial membrane potential, ATP, and GSH) (Figure 2D,E), and enhanced cellular senescence (Figure 2F,G; Figure S2B–F). Notably, co‐treatment with the miR‐103a‐3p inhibitor significantly alleviated these functional impairments (Figure 2B–G; Figure S2B–F). Consistently, Pyr‐EVs derived from macrophages transfected with a miR‐103a‐3p inhibitor showed a markedly reduced capacity to induce apoptosis, mitochondrial dysfunction, and cellular senescence in KGN cells (Figure S2G–R), further supporting EVs‐associated miR‐103a‐3p as an important functional mediator of the detrimental effects of Pyr‐EVs.

**FIGURE 2:**
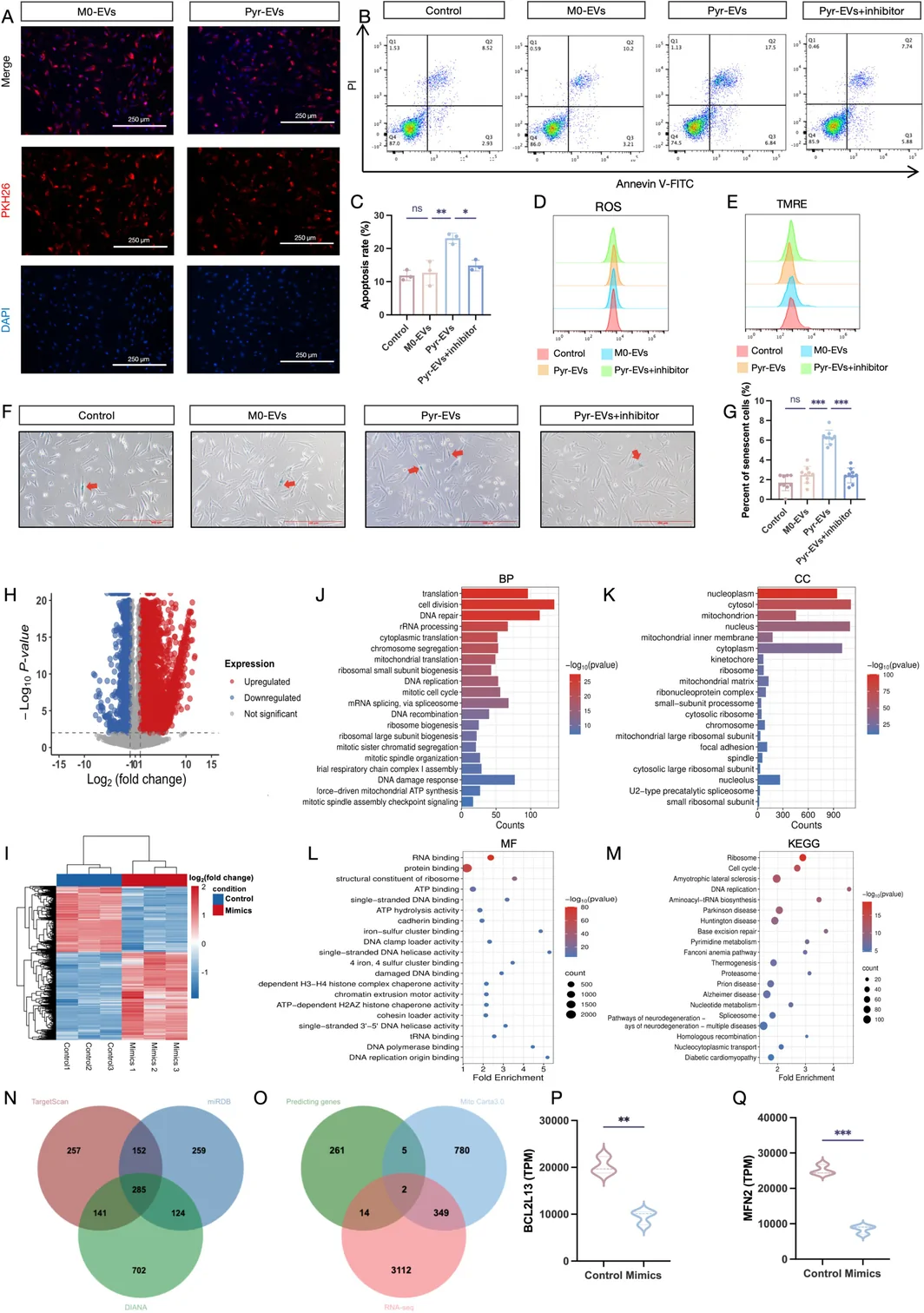
Pyr‐EVs induce mitochondrial dysfunction and apoptosis in KGN cells via miR‐103a‐3p and its downstream targets. (A) PKH26‐labeled EVs were incubated with KGN cells, and uptake was observed under fluorescence microscopy. (B, C) Flow cytometry analysis of apoptosis using Annexin V‐FITC/PI staining (*n* = 3). (D, E) Flow cytometry detection of cellular ROS levels (DHE staining) and quantitative measurement of mitochondrial membrane potential (*n* = 3). (F, G) SA‐β‐gal staining to assess cellular senescence (*n* = 9). (H) Volcano plot of DEmRNAs with criteria |log2FC| ≥ 1 and *p* < 0.01 (*n* = 3). (I) Heatmap of DEmRNAs; darker colors indicate greater differential expression (*n* = 3). (J–L) GO enrichment analysis of downregulated DEmRNAs, showing results for BP, CC, and MF, respectively. (M) KEGG pathway enrichment analysis of downregulated DEmRNAs. (N) Venn diagram of predicted miR‐103a‐3p target genes from DIANA, miRDB, and TargetScan databases. (O) Venn diagram intersecting the 285 predicted target genes with downregulated DEmRNAs and mitochondrial genes from MitoCarta3.0. (P, Q) RNA‐seq expression levels of MFN2 and BCL2L13 mRNAs. Data are presented as mean ± SD. Statistical significance was analyzed using one‐way ANOVA followed by Tukey's multiple‐comparison test, as appropriate. ^ns^ *p* > 0.05, *p < 0.05, **p < 0.01, ***p < 0.001.

Subsequently, KGN cells were transfected with miR‐103a‐3p mimics, and RNA‐seq was performed to identify downstream target genes. Compared with the control group, the miR‐103a‐3p mimic group (Mimics group) displayed 9558 differentially expressed mRNAs (DEmRNAs), with 6160 upregulated and 3398 downregulated based on the criteria of |log2(fold change)| ≥ 1 and adjusted *p‐value* (FDR) < 0.01 (Figure 2H,I). GO enrichment analysis revealed that the downregulated DEmRNAs were primarily involved in protein translation and ribosome biogenesis, cell cycle and mitotic regulation (e.g., chromosome segregation and spindle assembly), DNA replication and damage repair, as well as mitochondrial energy metabolism‐related processes (Figure 2J). Cellular component analysis indicated that the differential genes were mainly localized to nuclear and cytoplasmic structures (e.g., nucleoplasm, nucleus, cytosol), mitochondria and its inner membrane and matrix, as well as ribosomes and ribonucleoprotein complexes (Figure 2K). In terms of molecular function, the differential genes were enriched for RNA and protein binding, ribosomal structural components, ATP binding and hydrolysis activity, as well as functions related to DNA replication, repair, and chromatin remodeling (Figure 2L). KEGG pathway analysis further highlighted enrichment in Ribosome, Cell cycle, and DNA replication pathways (Figure 2M).

Additionally, by querying the DIANA, miRDB, and TargetScan databases, we intersected the predicted targets of miR‐103a‐3p and identified 285 potential downstream genes (Figure 2N). Further intersection of these 285 genes with downregulated DEmRNAs and mitochondrial genes from MitoCarta3.0 yielded two potential targets: *MFN2* and *BCL2L13* (Figure 2O–Q).

### 3.3 miR‐103a‐3p Levels in Human Follicular Fluid EVs Correlate With Ovarian Reserve and ART Outcomes

Clinical follicular fluid EVs were collected and characterized. EVs from the DOR group exhibited significantly larger particle size and higher concentration compared with the NOR group (Figure 3A–E), similar to Pyr‐EVs. miR‐103a‐3p expression was markedly elevated in DOR FF‐EVs and granulosa cells relative to NOR controls (Figure 3F,G). In DOR patients, granulosa cells displayed reduced ATP levels (Figure 3H) and increased ROS and MDA levels (Figure 3I,J). Additionally, expression of mitochondrial genes MFN2 and BCL2L13 at both mRNA and protein levels was significantly lower in DOR granulosa cells (Figure 3K–O). Apoptosis‐related factors, including BCL2 and steroidogenic markers FSH and CYP11A1, were decreased in DOR granulosa cells, whereas BAX and LHCGR levels were increased. SASP markers IL6 and IL8 mRNA, as well as p16 and p21 protein levels, were also elevated in DOR patients (Figure 3P–R and Figure S3A–H).

**FIGURE 3:**
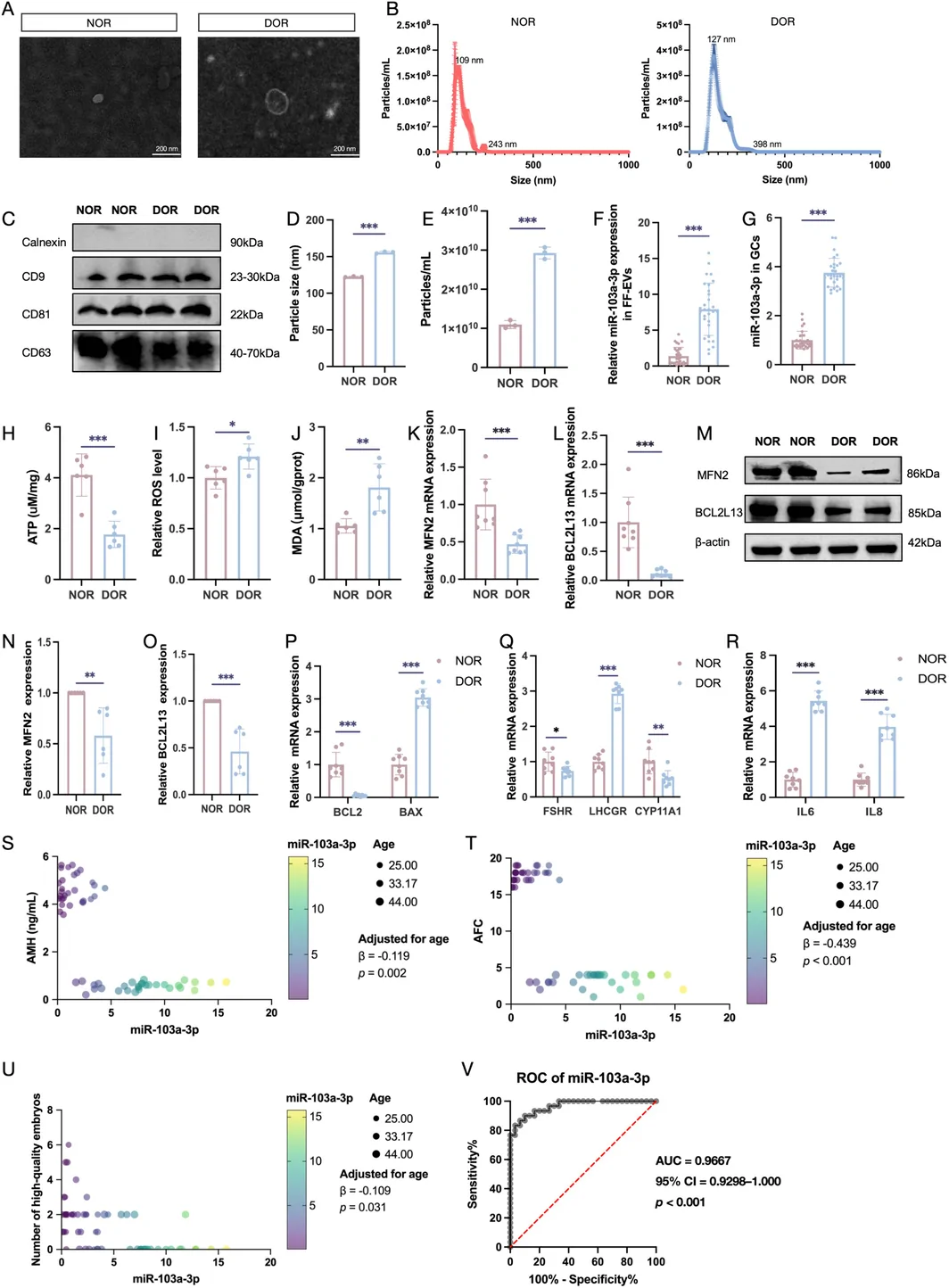
miR‐103a‐3p levels in human follicular fluid EVs correlate with ovarian reserve and ART outcomes. (A) TEM images showing the morphology of EVs in follicular fluid. (B) NTA analysis of EVs particle size (*n* = 3). (C) Western blot detection of EVs markers CD9, CD81, and CD63, with Calnexin as a negative control (*n* = 6). A subset of samples (*n* = 6 per group) was used due to limited cell availability, and these samples were randomly selected from the clinical cohort. (D, E) Comparison of EVs particle size and concentration between groups. (F, G) Relative expression of miR‐103a‐3p in FF‐EVs and granulosa cells from DOR and NOR patients (*n* = 30). (H–J) ATP, ROS, and MDA levels in granulosa cells (*n* = 6). (K, L) qRT‐PCR analysis of *MFN2* and *BCL2L13* mRNA expression (*n* = 8). (M–O) Western blot analysis of MFN2 and BCL2L13 protein expression (*n* = 6). A subset of samples (*n* = 6 per group) was used due to limited cell availability, and these samples were randomly selected from the clinical cohort. Quantified protein expression was normalized to β‐actin and expressed relative to the NOR group (set as 1.0). (P–R) qRT‐PCR analysis of apoptosis‐related genes (BCL2, BAX), steroidogenic markers (FSH, CYP11A1), and SASP factors (IL6, IL8) (*n* = 8). (S–U) Correlation analyses between FF‐EVs miR‐103a‐3p levels and patient AMH, AFC, and high‐quality embryos (*n* = 30). (V) ROC curve analysis between FF‐EVs miR‐103a‐3p levels and DOR. Data are presented as mean ± SD. Replication numbers are indicated in the corresponding panels. Statistical significance was determined using an unpaired two‐tailed Student's *t*‐test. ^ns^ *p* > 0.05, *p < 0.05, **p < 0.01, ***p < 0.001.

To evaluate whether age, Gn dose, and study group were independently associated with follicular fluid EVs‐derived miR‐103a‐3p expression, a multivariable linear regression model was constructed with miR‐103a‐3p expression as the dependent variable and age, Gn dose, and study group as independent variables. The results showed that only study group was independently associated with miR‐103a‐3p expression (*p* < 0.001), whereas neither Gn dose (*p* = 0.331) nor age (*p* = 0.887) showed a significant association (Figure S3I). After adjustment for age, miR‐103a‐3p expression was negatively associated with serum AMH levels (*β* = −0.119, *p* = 0.002), AFC (*β* = −0.439, *p* < 0.001), and the number of high‐quality embryos (*β* = −0.109, *p* = 0.031) (Figure 3S–U). Furthermore, miR‐103a‐3p expression was negatively associated with the number of retrieved oocytes (*β* = −0.455, *p* < 0.001), fertilized oocytes (*β* = −0.227, *p* = 0.033), and embryos (*β* = −0.183, *p* = 0.004), whereas no significant association was observed with the number of normally fertilized oocytes (*β* = −0.267, *p* = 0.094). In contrast, miR‐103a‐3p expression was positively associated with serum estradiol (E_2_) levels on the trigger day (*β* = 153, *p* < 0.001) (Supplementary Figure S3J–N). In addition, basal FSH, basal LH, basal E2, LH on the trigger day, and progesterone on the trigger day were not independently associated with miR‐103a‐3p expression (all *p* > 0.05; Figure S3O). ROC curve analysis further demonstrated that follicular fluid EVs‐derived miR‐103a‐3p effectively discriminated DOR from NOR, with an AUC of 0.9667 (95% CI: 0.9298–1.000, *p* < 0.001) (Figure 3V).

### 3.4 miR‐103a‐3p Induces Mitochondrial Dysfunction in Granulosa Cells Through Targeted Inhibition of MFN2 and BCL2L13

Figure 4A shows the predicted binding sites of the miR‐103a‐3p seed sequence in the 3′‐untranslated regions (3′‐UTRs) of *MFN2* and *BCL2L13* mRNAs, as identified by TargetScan. These fragments were cloned downstream of the luciferase coding sequence in a reporter plasmid. The dual‐luciferase reporter assay demonstrated that miR‐103a‐3p significantly reduced the luciferase activity of the *MFN2*‐WT and *BCL2L13*‐WT constructs, whereas luciferase activity remained unchanged when the predicted binding sites were mutated (Figure 4B,C). Overexpression of miR‐103a‐3p significantly decreased the mRNA and protein levels of MFN2 and BCL2L13 (Figure 4D–H), whereas inhibition of miR‐103a‐3p led to significant increases in their mRNA and protein expression (Figure 4D–H). These findings confirm that MFN2 and BCL2L13 are direct downstream targets of miR‐103a‐3p.

**FIGURE 4:**
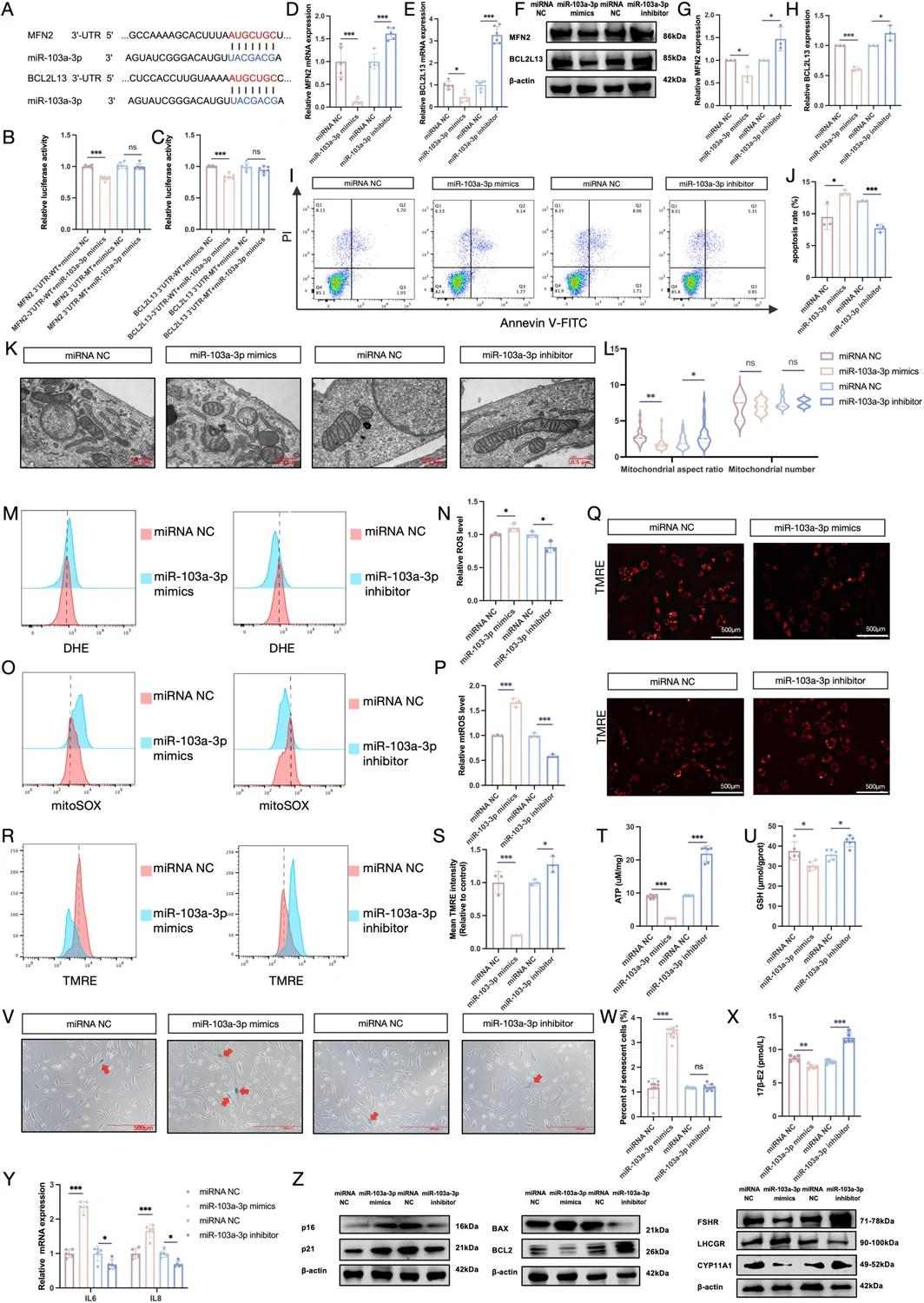
miR‐103a‐3p induces mitochondrial dysfunction in granulosa cells by targeting and inhibiting MFN2 and BCL2L13. (A) Predicted binding sites of the miR‐103a‐3p seed sequence in the 3′‐UTRs of *MFN2* and *BCL2L13* mRNAs. (B, C) Dual‐luciferase reporter assay (*n* = 6). (D, E) qRT‐PCR analysis of *MFN2* and *BCL2L13* mRNA expression (*n* = 5). (F–H) Western blot analysis of MFN2 and BCL2L13 protein expression (*n* = 3). Quantified protein expression was normalized to β‐actin and expressed relative to the miRNA NC group (set as 1.0). (I, J) Flow cytometry (Annexin V‐FITC/PI double staining) to assess apoptosis (*n* = 3). (K, L) TEM analysis of mitochondrial morphology; mitochondrial aspect ratio (length/width) (*n* = 30) and mitochondrial number per cell (*n* = 6). (M, N) Flow cytometry (DHE staining) to detect intracellular ROS levels (*n* = 3). (O, P) Flow cytometry (MitoSOX staining) to measure mitochondrial ROS levels (*n* = 3). (Q–S) TMRE staining and flow cytometry for mitochondrial membrane potential quantification (*n* = 3). (T, U) ATP and GSH level determination (*n* = 5). (V, W) SA‐β‐gal staining to assess cellular senescence (*n* = 9). (X) Measurement of 17β‐estradiol levels in the cell supernatant (*n* = 5). (Y) qRT‐PCR analysis of SASP factors (*IL6* and *IL8*) mRNA expression (*n* = 5). (Z) Western blot detection of p16, p21, BAX, BCL2, FSHR, LHCGR, and CYP11A1 protein expression (*n* = 3). Data are presented as mean ± SD. Replication numbers are indicated in the corresponding panels. Statistical significance was determined using an unpaired two‐tailed Student's *t*‐test. ^ns^ *p* > 0.05, *p < 0.05, **p < 0.01, ***p < 0.001.

In addition, miR‐103a‐3p mimics markedly promoted granulosa cell apoptosis (Figure 4I,J), while the miR‐103a‐3p inhibitor significantly reduced the apoptosis rate. miR‐103a‐3p overexpression also exacerbated oxidative stress injury, as evidenced by mitochondrial morphological alterations characterized by a decreased aspect ratio (Figure 4K,L), suggesting enhanced mitochondrial fragmentation, along with increased ROS, mtROS, and MDA levels (Figure 4M–P; Figure S6B), and reduced mitochondrial membrane potential, ATP production, and GSH levels (Figure 4Q–U). Conversely, opposite trends were observed in granulosa cells treated with the miR‐103a‐3p inhibitor (Figure 4K–Q).

Furthermore, cellular senescence was increased in the miR‐103a‐3p mimics group compared with the miRNA NC group (Figure 4V,W). The concentration of E_2_ in the cell supernatant was significantly reduced in the miR‐103a‐3p mimics group but increased in cells transfected with the miR‐103a‐3p inhibitor (Figure 4U). In addition, expression of SASP‐related cytokines (*IL6* and *IL8* mRNAs) was significantly upregulated in the miR‐103a‐3p mimics group (Figure 4Y), along with elevated p16 and p21 protein levels (Figure 4Z; Figure S4C–N). Expression of steroidogenesis‐related factors, including CYP11A1, FSHR, and LHCGR, was also altered (Figure 4Z; Figure S4C–N).

Collectively, these results suggest that miR‐103a‐3p overexpression induces apoptosis, mitochondrial dysfunction, impaired E_2_ secretion, and cellular senescence in granulosa cells.

### 3.5 Overexpression of MFN2 and/or BCL2L13 Reverses miR‐103a‐3p‐Induced Impairment of Granulosa Cell Dysfunction Under Oxidative Stress Conditions

To mimic the mitochondrial dysfunction induced by Pyr‐EVs in vitro, we exposed granulosa cells to 3‐NP to induce oxidative stress, providing a controllable model for mechanistic analysis. OS in granulosa cells was induced using 3‐NP, an inhibitor of mitochondrial complex II that promotes mitochondrial ROS generation and release. According to a previous study and CCK‐8 assay results, the optimal 3‐NP concentration was determined to be 10 mM (Wang, Yang, et al. 2023) (Figure S5A,B). The results indicated that the OS model was successfully established in granulosa cells. Compared with the control group, the OS group exhibited increased apoptosis (Figure S5C,D), decreased ATP, GSH levels, mitochondrial membrane potential, and elevated cellular ROS, mtROS, MDA levels, and mitochondrial swelling (Figure S5E–O).

MFN2 is known to play a critical role in maintaining mitochondrial function in granulosa cells, and its reduced expression is considered a potential indicator of decreased ovarian reserve, cellular aging, and assisted reproduction failure (Wang et al. 2019; Zhang, Bener, et al. 2019). BCL2L13 functions as a mitochondrial autophagy receptor that regulates mitochondrial dynamics and mass (Wang, Chen, et al. 2023). To determine whether MFN2 and BCL2L13 participate in the miR‐103a‐3p‐mediated regulation of granulosa cell dysfunction, we overexpressed *MFN2* and *BCL2L13* in miR‐103a‐3p mimics‐transfected granulosa cells under oxidative stress conditions. The results showed that exogenous overexpression of MFN2, BCL2L13, or their combined overexpression (M/B‐OE) effectively rescued the detrimental effects induced by miR‐103a‐3p mimics. Specifically, these interventions significantly reduced apoptosis (Figure 5A,C) and restored mitochondrial morphology, as evidenced by an increased mitochondrial length‐to‐width ratio and enhanced filamentous mitochondrial networks (Figure 5B,D). Moreover, overexpression of MFN2, BCL2L13, or both markedly reduced intracellular ROS, mtROS, and MDA levels while restoring GSH content, mitochondrial membrane potential, and ATP production (Figure 5E–J; Figure S5Q–S). Notably, combined overexpression of MFN2 and BCL2L13 resulted in a significantly greater recovery of mitochondrial membrane potential and ATP levels than either single‐gene rescue alone, whereas no additional improvement was observed for ROS, mtROS, MDA, or GSH, suggesting that the cooperative effects of the two targets are primarily associated with mitochondrial bioenergetic function. These treatments also significantly attenuated cellular senescence (Figure 5K,M,N). Given that mitochondria are the primary site of E_2_ biosynthesis, restoration of MFN2 and/or BCL2L13 expression also increased E_2_ secretion into the culture supernatant (Figure 5L), likely through recovery of mitochondrial function. Furthermore, the mRNA and protein expression levels of mitochondrial function‐, apoptosis‐, and steroidogenesis‐related molecules were correspondingly restored (Figure 5O; Figure S5V–AH). Collectively, these findings demonstrate that restoration of MFN2 and BCL2L13 effectively counteracts miR‐103a‐3p‐induced mitochondrial dysfunction and granulosa cell injury, while their combined restoration provides additional benefits for mitochondrial bioenergetic recovery.

**FIGURE 5:**
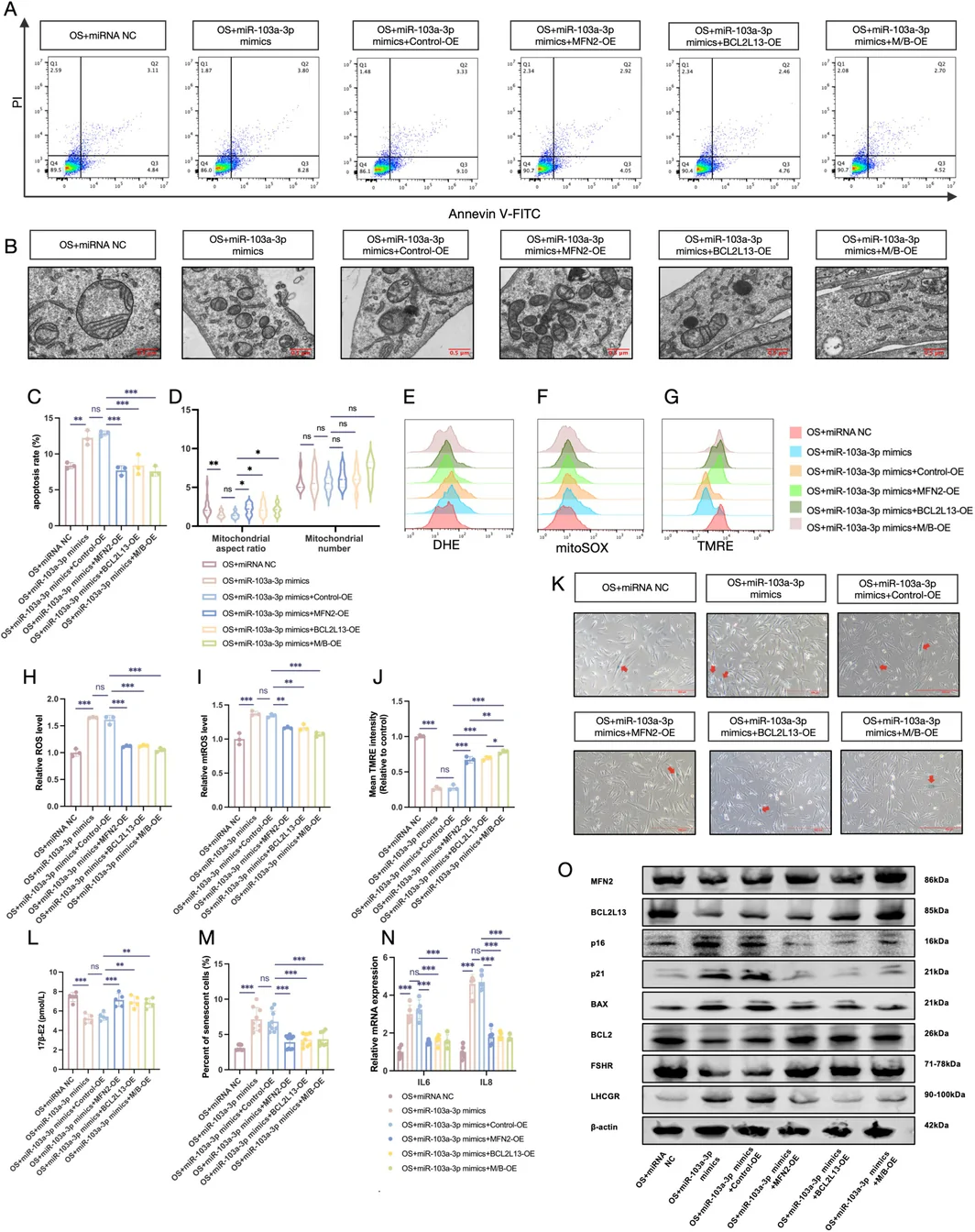
Overexpression of MFN2 and/or BCL2L13 reverses miR‐103a‐3p‐induced impairment of granulosa cell dysfunction under oxidative stress conditions. (A) Flow cytometry (Annexin V‐FITC/PI double staining) for apoptosis detection (*n* = 3). (B) TEM analysis of mitochondrial morphology. (C) Statistics on the percentage of apoptotic cells (*n* = 3). (D) Statistics on TEM analysis; mitochondrial aspect ratio (length/width) (*n* = 30) and number of mitochondria per cell (*n* = 6). (E–J) Flow cytometry (DHE staining) for cellular ROS levels (*n* = 3); flow cytometry (MitoSOX staining) for mitochondrial ROS (mtROS) levels (*n* = 3); TMRE staining and flow cytometry for mitochondrial membrane potential quantification (*n* = 3). (K) SA‐β‐gal staining for cellular senescence assessment (*n* = 9). (L) Measurement of 17β‐estradiol levels in the cell supernatant (*n* = 5). (M) Statistics on the percentage of senescent cells (SA‐β‐gal staining, *n* = 9). (N) qRT‐PCR analysis of SASP‐related cytokines (*IL6* and *IL8*) mRNA expression (*n* = 5). (O) Western blot analysis of MFN2, BCL2L13, p16, p21, BAX, BCL2, FSHR, LHCGR protein expression (*n* = 3). Data are presented as mean ± SD. Statistical significance was analyzed using one‐way ANOVA followed by Tukey's multiple‐comparison test, as appropriate. ^ns^ *p* > 0.05, *p < 0.05, **p < 0.01, ***p < 0.001.

Collectively, these findings indicate that *MFN2* and *BCL2L13* are regulated by miR‐103a‐3p, and that their overexpression alleviates miR‐103a‐3p‐induced granulosa cell dysfunction under oxidative stress by restoring mitochondrial dynamics, reducing apoptosis, and improving steroidogenic capacity.

### 3.6 Inhibition of miR‐103a‐3p Ameliorates Oxidative Stress‐Induced Ovarian Injury in Mice

To mimic oxidative stress conditions observed in aging or pathological ovaries, mice were exposed to oxidative stress, which phenocopies the mitochondrial dysfunction induced by Pyr‐EVs in vitro. miR‐103a‐3p was silenced in mouse ovaries via intraperitoneal injection of an AAV‐based miR‐103a‐3p inhibitor. Preliminary experiments were conducted to determine the optimal AAV dosage and administration timing (Figure S6A,B). Exposure to oxidative stress did not significantly affect body weight (Figure S6C). However, histological analysis revealed that the proportions of primordial follicles, primary follicles, and corpus lutea were markedly reduced, whereas atretic follicles were significantly increased in the OS group (Figure 6A,B). In addition, oxidative stress caused a substantial reduction in serum E_2_ and AMH levels and a concomitant elevation in FSH and LH levels (Figure 6C–F), accompanied by disruption of the estrous cycle (Figure S6D,E). Conversely, in mice treated with the AAV‐miR‐103a‐3p inhibitor, E_2_, AMH, and the percentages of primordial and primary follicles were significantly increased, while FSH, LH, and atretic follicle ratios were significantly decreased under oxidative stress conditions (Figure 6A–F).

**FIGURE 6:**
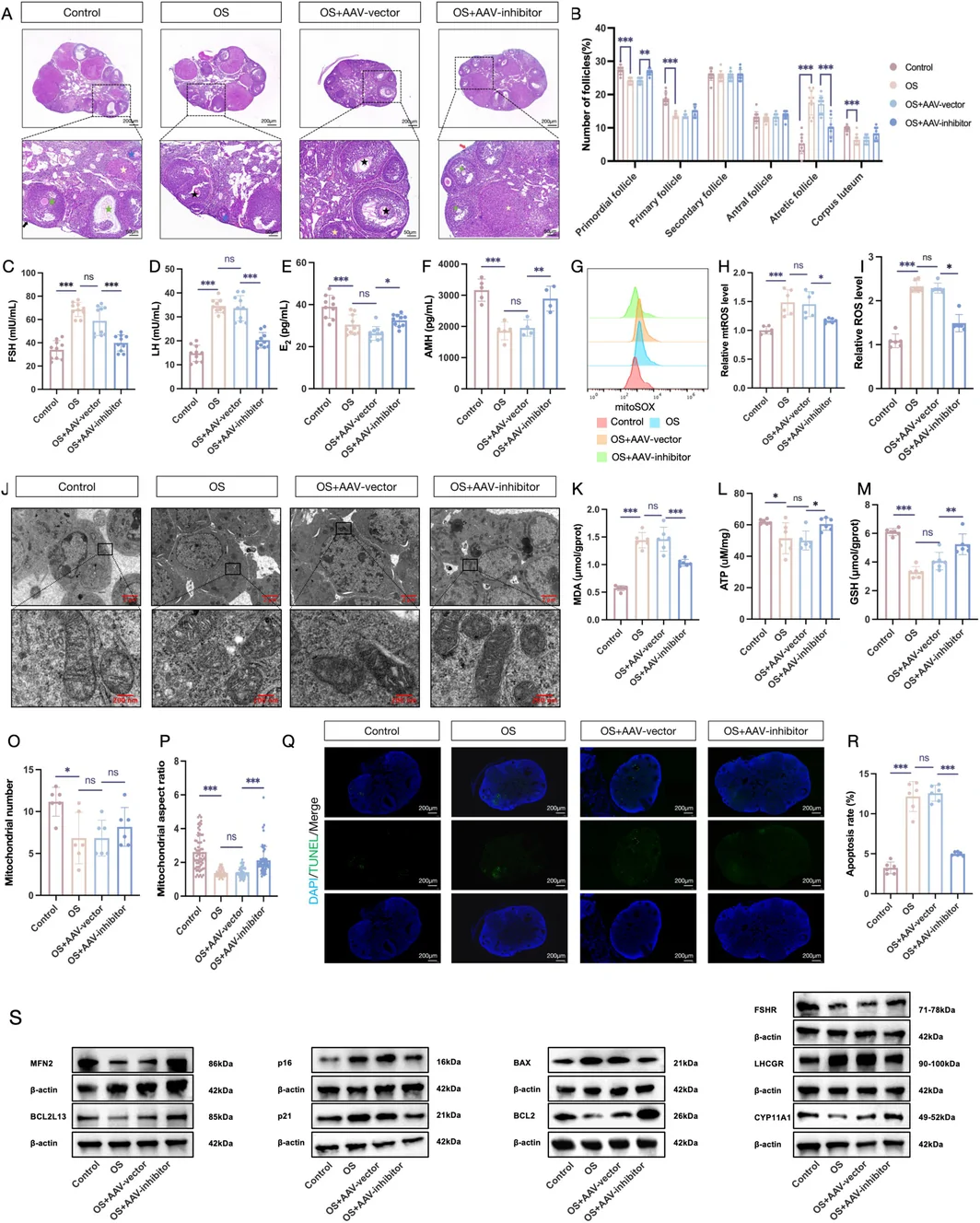
Inhibition of miR‐103a‐3p ameliorates oxidative stress‐induced ovarian injury in mice. (A) H&E staining showing ovarian morphology (120× and 400× magnification); black stars: Atretic follicles; yellow stars: Corpus lutea; blue stars: Secondary follicles; red arrows: Primordial follicles; black arrows: Primary follicles. (B) Quantification of follicle proportions at each developmental stage (*n* = 10). (C–F) ELISA analysis of serum FSH, LH, E_2_, and AMH levels in mice (*n* = 10). (G, H) Flow cytometry (MitoSOX staining) to assess mtROS levels (*n* = 6). (I) DHE staining to measure cellular ROS levels (*n* = 6). (J) TEM observation of granulosa cell mitochondrial morphology (*n* = 6). (K–M) Measurement of MDA, ATP, and GSH levels (*n* = 6). (O, P) Quantification of mitochondrial aspect ratio (length/width) (*n* = 30) and number of mitochondria per cell (*n* = 6). (Q, R) TUNEL staining to assess apoptosis in ovarian tissue (*n* = 6). (S) Western blot analysis of MFN2, BCL2L13, p16, p21, BAX, BCL2, FSHR, LHCGR, and CYP11A1 protein expression in ovarian tissue (*n* = 3). Data are presented as mean ± SD. Statistical significance was analyzed using one‐way ANOVA followed by Tukey's multiple‐comparison test, as appropriate. ^ns^ *p* > 0.05, *p < 0.05, **p < 0.01, ***p < 0.001.

Furthermore, miR‐103a‐3p inhibition ameliorated oxidative damage in the ovary, as evidenced by reduced mtROS, ROS, and MDA levels, along with increased ATP and GSH activity (Figure 6G–I,K–M). TEM showed improved mitochondrial morphology, with a notable reduction in fragmentation and swelling (Figure 6J,O,P). Apoptosis in ovarian granulosa cells was evaluated using the TUNEL assay, which demonstrated a significant reduction in apoptotic cells in the AAV‐inhibitor group compared with the AAV‐vector group, suggesting that miR‐103a‐3p inhibition suppresses granulosa cell apoptosis under oxidative stress (Figure 6Q,R). Additionally, miR‐103a‐3p inhibition significantly increased both the mRNA and protein expression of MFN2 and BCL2L13 and restored the expression of apoptosis‐, senescence‐, and steroidogenesis‐related genes and proteins in ovarian tissue (Figure 6S; Figure S6F–W).

Collectively, these results indicate that miR‐103a‐3p inhibition alleviates oxidative stress‐induced ovarian dysfunction by restoring mitochondrial integrity, reducing apoptosis, and normalizing steroid hormone synthesis in granulosa cells.

## 4 Discussion

This study provides mechanistic insight into how pyroptotic macrophages may promote mitochondrial dysfunction in granulosa cells and contribute to ovarian aging through immuno‐inflammatory regulation and intercellular communication. Importantly, our findings extend previous miRNA studies in DOR by identifying a distinct cellular origin and mode of action for miR‐103a‐3p. While most reported ovarian aging‐associated miRNAs are produced within granulosa cells or oocytes and function through cell‐autonomous mechanisms, we demonstrate that miR‐103a‐3p originates from pyroptotic macrophages and is transferred to granulosa cells through extracellular vesicles. These findings support the existence of a previously unrecognized immune‐to‐ovarian communication axis linking inflammatory macrophage activation to mitochondrial dysfunction and ovarian decline.

Through single‐cell RNA sequencing analysis, this study revealed that pyroptosis‐related signaling in ovarian macrophages is significantly enhanced with aging, indicating a shift of the ovarian immune microenvironment from a homeostatic state toward a pro‐inflammatory phenotype. Previous studies have shown that pyroptosis, as an inflammatory form of programmed cell death, amplifies local inflammatory responses through the release of cytokines such as IL‐1β and IL‐18 (Rao et al. 2022). However, our findings further suggest that pyroptotic macrophages not only mediate inflammation via cytokine secretion but also participate in intercellular communication through the release of EVs, thereby potentially exerting more sustained regulatory effects on the ovarian microenvironment. Although our data support a role for pyroptotic macrophages in granulosa cell dysfunction, they do not establish whether macrophage pyroptosis represents an initiating event during DOR progression or develops secondary to ovarian injury. These processes may instead constitute a positive feedback loop that amplifies ovarian inflammation and dysfunction. In addition, the upstream regulatory mechanisms driving miR‐103a‐3p enrichment in pyroptotic macrophages warrant further investigation. Pyroptosis is closely associated with activation of inflammasome‐related signaling pathways, particularly NLRP3 and NF‐κB, which play central roles in inflammatory responses (Kinoshita et al. 2015). Previous studies have shown that miR‐103a‐3p is involved in NF‐κB‐related inflammatory regulation, suggesting a potential association between inflammatory activation and miRNA expression (Li, Liang, et al. 2020, 1; Lu et al. 2019). Whether these pathways directly regulate miR‐103a‐3p expression and its EVs‐mediated loading in pyroptotic macrophages remains to be determined. Together, these observations raise the possibility of an inflammation‐driven miRNA reprogramming process underlying EVs‐mediated intercellular signaling.

At the mechanistic level, we found that EVs derived from pyroptotic macrophages were efficiently internalized by granulosa cells, leading to mitochondrial dysfunction, apoptosis, and cellular senescence. miRNA sequencing identified a significant enrichment of miR‐103a‐3p in Pyr‐EVs, which was further supported as a key effector through functional assays. Importantly, inhibition of miR‐103a‐3p markedly attenuated Pyr‐EVs‐induced granulosa cell injury, supporting its functional contribution to EVs‐mediated cellular damage. Collectively, these findings provide functional evidence that EVs‐associated miR‐103a‐3p is an important mediator of granulosa cell injury induced by Pyr‐EVs.

Oxidative stress is a central contributor to ovarian aging and premature follicular atresia, primarily through excessive ROS accumulation and impaired antioxidant defenses (Gorrini et al. 2013). As the major somatic cells supporting follicular development and oocyte maturation, granulosa cells are particularly vulnerable to oxidative damage (Chaube et al. 2005). Disruption of redox homeostasis leads to mitochondrial dysfunction, activation of apoptotic pathways, and impaired steroidogenesis, ultimately compromising oocyte quality and developmental competence (Chaube et al. 2005; Chen et al. 2024). In this context, mitochondrial integrity in granulosa cells is considered an important determinant of ovarian reserve (Wasielak‐Politowska and Kordowitzki 2022). Our findings suggest that EVs‐mediated delivery of miR‐103a‐3p exacerbates oxidative stress‐associated mitochondrial dysfunction, thereby promoting granulosa cell injury. These findings place EVs‐associated miRNA signaling within the broader framework of oxidative stress‐related ovarian pathology.

In recent years, increasing evidence has highlighted the regulatory roles of miRNAs in ovarian development, ovulation, luteinization, apoptosis, and oxidative stress. For example, miR‐484 impairs ovarian function by suppressing SESN2 (Wang, Yang, et al. 2023), miR‐642a‐5p targets FOXO1 and contributes to mitochondrial dysfunction (Zhu et al. 2024), and miR‐138‐5p promotes granulosa cell apoptosis through SIRT1 downregulation (Yu et al. 2023). Notably, these studies primarily focus on miRNAs that are intrinsically expressed within ovarian cells and are induced as downstream responses to cellular stress. In contrast, our findings suggest a distinct regulatory paradigm in which miR‐103a‐3p is derived from pyroptotic macrophages and delivered to granulosa cells through EVs‐mediated intercellular communication. Thus, miR‐103a‐3p may function not only as a regulator of intracellular stress responses but also as an immune‐derived signaling molecule contributing to ovarian dysfunction. This non‐cell‐autonomous mechanism expands the current understanding of how immune cell‐derived EVs‐miRNAs may participate in ovarian aging and DOR.

Notably, in clinical samples, we found that miR‐103a‐3p was significantly elevated in follicular fluid‐derived EVs from patients with DOR and negatively correlated with AMH levels, AFC, and embryo developmental outcomes. These findings support the potential of EVs‐associated miR‐103a‐3p as a follicular fluid indicator of ovarian reserve and reproductive outcomes; however, its value as a clinical biomarker requires validation in larger, prospective cohorts. MFN2 plays a vital role in maintaining mitochondrial dynamics, steroidogenesis, and other essential cellular functions (Chen et al. 2003; Mengus et al. 2022), and its reduced expression has been closely linked to ovarian dysfunction and premature follicular failure (Wang et al. 2019; Zhang, Bener, et al. 2019; Kütük et al. 2024). Preservation of MFN2 function in granulosa cells has been shown to inhibit oxidative stress, cellular senescence, and apoptosis (Li et al. 2025; Hou et al. 2024; Xing et al. 2025). Meanwhile, BCL2L13, a mitophagy receptor, regulates mitochondrial homeostasis by facilitating the removal of damaged mitochondria (Wang, Chen, et al. 2023; Otsu et al. 2015; Meng et al. 2021). Our functional data indicate that miR‐103a‐3p overexpression promotes mitochondrial fragmentation, impaired mitophagy, and excessive ROS accumulation through the combined suppression of MFN2 and BCL2L13, leading to granulosa cell apoptosis, senescence‐like changes, and reduced estrogen secretion. These findings support a mechanistic link between mitochondrial dysfunction and impaired ovarian function. Furthermore, co‐restoration of *MFN2* and *BCL2L13* resulted in a greater recovery of mitochondrial membrane potential and ATP production than restoration of either gene alone, supporting their cooperative roles in maintaining mitochondrial bioenergetics. In contrast, co‐rescue did not provide additional improvement in oxidative stress‐related parameters, suggesting that restoration of either target alone is sufficient to largely re‐establish redox homeostasis under the present experimental conditions. Importantly, this mechanism differs from previously reported ovarian miRNAs that primarily regulate apoptosis‐ or antioxidant‐related pathways. By simultaneously targeting MFN2 and BCL2L13, miR‐103a‐3p may connect immune‐derived EVs signaling with mitochondrial dynamics and mitophagy, providing one possible mechanistic explanation for how inflammatory microenvironmental changes contribute to progressive ovarian dysfunction.

From a translational perspective, EVs‐associated miR‐103a‐3p shows potential as a candidate follicular fluid indicator and a therapeutic target. Follicular fluid‐derived EVs can be readily obtained during assisted reproductive procedures, and their cargo is relatively stable, supporting their potential utility for translational research. The observed correlations between miR‐103a‐3p levels and ovarian reserve indicators (e.g., AMH and AFC), as well as embryo developmental outcomes, warrant further investigation in larger clinical cohorts. In addition, our in vivo findings suggest that inhibition of miR‐103a‐3p alleviates mitochondrial dysfunction and improves ovarian function, supporting its potential as a therapeutic target. Therapeutic approaches such as antagomirs, AAV‐mediated inhibition, or EVs‐based delivery systems may enable targeted modulation of miR‐103a‐3p activity. However, challenges including delivery specificity, off‐target effects, and long‐term safety will need to be addressed before clinical translation.

Notably, our in vivo experiments demonstrated that inhibition of miR‐103a‐3p improved mitochondrial structure and function in mouse ovaries, restored follicular development, and partially normalized hormonal balance, as reflected by reduced follicular atresia and increased E_2_ and AMH levels. These findings further support the functional contribution of miR‐103a‐3p to ovarian dysfunction and suggest that its inhibition may have therapeutic potential. Compared with conventional antioxidant therapies, miRNA‐targeted interventions may act at an upstream regulatory level and therefore provide a more mechanism‐based therapeutic strategy (Diener et al. 2022). Nevertheless, the feasibility, specificity, and long‐term safety of miR‐103a‐3p‐targeted approaches require further investigation before clinical application.

This study has several limitations that should be acknowledged. An important limitation is the lack of cell‐type specificity associated with systemic AAV‐mediated delivery. Although our in vivo experiments were designed to target granulosa cells, systemically administered AAV vectors may also transduce other ovarian cell types, including oocytes. Therefore, the observed improvements in ovarian function cannot be attributed exclusively to granulosa cells, and contributions from other ovarian cell populations cannot be excluded. Nevertheless, our in vitro findings consistently support a direct effect of miR‐103a‐3p on granulosa cells. Future studies employing cell type‐specific genetic or viral targeting strategies will be required to define the relative contributions of granulosa cells and other ovarian cell types more precisely. Furthermore, although our data support an association between macrophage pyroptosis, EVs‐mediated miR‐103a‐3p transfer, and granulosa cell dysfunction, they do not establish the temporal sequence of these events in vivo. Longitudinal studies incorporating lineage tracing or time‐course analyses will be necessary to determine whether macrophage pyroptosis precedes, accompanies, or follows ovarian injury during DOR progression. In addition, EVs carry a variety of bioactive molecules, including proteins and lipids, whose potential contributions to granulosa cell dysfunction remain to be elucidated. Moreover, the dynamic changes of pyroptotic macrophages and their interactions with other immune cell populations during DOR progression remain incompletely understood. Future studies integrating single‐cell multi‐omics and in vivo tracing approaches will help define EVs‐mediated intercellular communication within the ovarian microenvironment more comprehensively. Furthermore, the downstream signaling mechanisms linking MFN2 and BCL2L13 dysregulation to mitochondrial dynamics and mitophagy were not comprehensively investigated in the present study. Future studies focusing on the molecular crosstalk between these two mitochondrial regulators and their downstream signaling pathways will provide a more complete understanding of the miR‐103a‐3p regulatory network. Finally, this study was based on a 3‐NP‐induced acute oxidative stress model, which does not fully recapitulate the chronic and heterogeneous progression of human DOR. Moreover, 3‐NP primarily induces mitochondrial dysfunction through chemical inhibition and therefore may not fully reflect the complex pathological processes underlying EVs‐mediated mitochondrial impairment in vivo. Accordingly, our findings should be interpreted within the context of this experimental model. Further validation in complementary animal models and experimental systems that more closely resemble the clinical pathophysiology of DOR will be important. In particular, chronic or etiologically distinct models, such as chemotherapy‐induced premature ovarian insufficiency or naturally aged mice, may help determine the broader applicability of the proposed mechanism. Collectively, these studies will strengthen the translational relevance and generalizability of our findings.

## 5 Conclusion

In summary, this study identifies a molecular mechanism by which pyroptotic macrophages may contribute to ovarian dysfunction through EVs‐mediated delivery of miR‐103a‐3p, leading to suppression of MFN2 and BCL2L13 and disruption of mitochondrial homeostasis in granulosa cells. These findings expand the current understanding of immune–ovarian communication in DOR and support the potential of EVs‐associated miR‐103a‐3p as a candidate follicular fluid indicator and therapeutic target, while warranting further validation in additional experimental models and clinical studies.

## Author Contributions

Wenhan Ju was one of the major contributors to writing the manuscript. Wenhan Ju, Xin Xin, and Yanli Xiao performed most experiments and interpreted the data. Wenhan Ju analyzed the data and prepared the figures and manuscript draft. Liwen Song, Jinfu Zhang, and Shan Xiang made substantial contributions to the conception and design of the work. All authors read and approved the final manuscript.

## Funding

This study was supported by the National Key R&D Program of China (2024YFC3505800), the Natural Science Foundation of Shandong Province of China (ZR2023MH112), and the Shenzhen Science and Technology Program (JCY20240813163012016), Guangdong Basic and Applied Basic Research Foundation (2025A1515010813).

## Ethics Statement

Our research ethics approval was obtained from the Reproductive Medicine Ethics Committee of the Reproductive Medical Center at the affiliated Hospital of Shandong University of traditional Chinese medicine (ref approval no. SDTCM20180130, January 13, 2018), which is in accordance with the Declaration of Helsinki. In addition, all participants signed written informed consent. The study was registered with the Chinese Clinical Trial Register (Registration number: ChiCTR1800019798, https://www.chictr.org.cn/index.html). All animal procedures and experimental protocols were approved by the Laboratory Animal Management and Use Committee of Tongren Hospital, Shanghai Jiao Tong University School of Medicine (Approval No. AF/SQ‐08/04.0).

## Consent

The use of human clinical samples and the publication of related data were approved by Reproductive Medicine Ethics Committee of the Reproductive Medical Center at the affiliated Hospital of Shandong University of traditional Chinese medicine, and written informed consent was obtained from all participants prior to sample collection.

## Conflicts of Interest

The authors declare no conflicts of interest.

## Supporting information


**Supplementary Table 1:** The baseline characteristics of the clinical patients.

**Table S2:** Primers used for quantitative real‐time polymerase chain reaction.

**Figure S1:** Single‐cell analysis reveals enhanced pyroptosis of macrophages in aged ovaries and identifies pyroptotic macrophage. (A–D) Expression levels of *PYCARD*, *CASP1*, *GSDMD*, and *IL‐1B* in macrophages across groups derived from the single‐cell RNA‐seq dataset GSE267315. (E, F) Measurement of Caspase‐1 p20 and GSDMD‐N protein expression (*n* = 3). Quantified protein expression was normalized to β‐actin and expressed relative to the M0 group (set as 1.0). Data are presented as mean ± SD. Statistical significance was determined using an unpaired two‐tailed Student's *t*‐test. ^ns^ *p* > 0.05, *p < 0.05, **p < 0.01, ***p < 0.001.
**Figure S2:** Pyr‐EVs induce mitochondrial dysfunction and apoptosis in KGN cells. (A) qRT‐PCR analysis of miR‐103a‐3p levels (*n* = 6). (B–F) Measurement of ROS, TMRE, ATP, MDA, and GSH levels (*n* = 5). (G) qRT‐PCR analysis of miR‐103a‐3p levels (*n* = 6). (H, I) Flow cytometry (Annexin V‐FITC/PI double staining) to assess apoptosis (*n* = 3). (J, K) Flow cytometry (DHE staining) to detect intracellular ROS levels (*n* = 3). (L, M) TMRE staining and flow cytometry for mitochondrial membrane potential quantification (*n* = 3). (N–P) Measurement of ATP, MDA, and GSH levels (*n* = 5). (Q, R) SA‐β‐gal staining to assess cellular senescence (*n* = 9). Data are presented as mean ± SD. Statistical significance was determined using an unpaired two‐tailed Student's *t*‐test for comparisons between two groups and one‐way ANOVA followed by Tukey's multiple comparisons test for comparisons among four groups. ^ns^ *p* > 0.05, *p < 0.05, **p < 0.01, ***p < 0.001.
**Figure S3:** miR‐103a‐3p levels in human follicular fluid EVs correlate with ovarian reserve and ART outcomes. (H) Western blot analysis of BCL2, BAX, FSHR, LHCGR, CYP11A1, p21 and p16 protein expression in follicular fluid granulosa cells from DOR and NOR patients (*n* = 3). Quantified protein expression was normalized to β‐actin and expressed relative to the NOR group (set as 1.0). Statistical significance was determined using the Mann–Whitney *U* test for comparisons between two groups. (I) Multiple linear regression analyses between FF‐EVs miR‐103a‐3p levels and Gn dose, age, group. (J–N) Correlation of miR‐103a‐3p expression levels in FF‐EVs with trigger‐day E_2_, retrieved oocytes, fertilized oocytes, normal fertilized oocytes, embryos, bFSH, bLH, bE_2_, and trigger‐day LH, trigger‐day progesterone levels. ^ns^ *p* > 0.05, *p < 0.05, **p < 0.01, ***p < 0.001.
**Figure S4:** miR‐103a‐3p induces mitochondrial dysfunction in granulosa cells by targeting and inhibiting MFN2 and BCL2L13 qRT‐PCR analysis of miR‐103a‐3p levels (*n* = 6). (B) Measurement of MDA levels (*n* = 5). (C–G) qRT‐PCR analysis of *BCL2, BAX, FSHR, LHCGR*, and *CYP11A1* mRNA expression (*n* = 5). (H‐N) Western blot detection of BCL2, BAX, FSHR, LHCGR, CYP11A1, p16 and p21 protein expression (*n* = 3). Quantified protein expression was normalized to β‐actin and expressed relative to the miRNA NC group (set as 1.0). Statistical significance was determined using an unpaired two‐tailed Student's *t*‐test or Mann–Whitney *U* test for comparisons between two groups, depending on data normality. ^ns^ *p* > 0.05, *p < 0.05, **p < 0.01, ***p < 0.001.
**Figure S5:** Overexpression of MFN2 and BCL2L13 reverses miR‐103a‐3p‐induced impairment of granulosa cell dysfunction under oxidative stress conditions. KGN cells were treated with varying concentrations of 3‐NP for different durations, and cell viability was assessed using the CCK‐8 assay (*n* = 3). (B) KGN cells were exposed to different concentrations of 3‐NP for 24 h, and cell viability was measured using the CCK‐8 assay (*n* = 3). (C, D) Flow cytometry (Annexin V‐FITC/PI double staining) to assess apoptosis (*n* = 3). (E–G) Measurement of ATP, GSH, and MDA levels (*n* = 5). (H–M) Flow cytometry (DHE staining, MitoSOX staining, TMRE staining) to detect intracellular ROS, mitochondrial ROS, mitochondrial membrane potential quantification (*n* = 3). (N‐O) TEM analysis of mitochondrial morphology; mitochondrial aspect ratio (length/width) (*n* = 30) and mitochondrial number per cell (*n* = 6). (P) qRT‐PCR analysis of miR‐103a‐3p levels (*n* = 6). (Q–S) Measurement of ATP, GSH, and MDA levels (*n* = 5). (T–Z) qRT‐PCR analysis of *MFN2, BCL2L13, BCL2, BAX, FSHR, LHCGR*, and *CYP11A1* mRNA expression (*n* = 5). (AA‐AH) Western blot detection of MFN2, BCL2L13, BAX, BCL2, FSHR, LHCGR, p16 and p21 protein expression (*n* = 3). Quantified protein expression was normalized to β‐actin and expressed relative to the OS+miRNA NC group (set as 1.0). Data are presented as mean ± SD. Statistical significance was analyzed using one‐way ANOVA followed by Tukey's multiple‐comparison test, as appropriate. ^ns^ *p* > 0.05, *p < 0.05, **p < 0.01, ***p < 0.001.
**Figure S6:** Inhibition of miR‐103a‐3p ameliorates oxidative stress‐induced ovarian injury in mice. Fluorescence co‐localization analysis confirmed the distribution of AAV in mouse ovarian cells 4 weeks after intraperitoneal injection. (B)Western blot detection of Luciferase protein expression (*n* = 3). Since Luciferase is inserted exogenously via the AAV vector, it can serve as a reporter for the inhibitory effect of miR‐103a‐3p. (C) Comparison of body weight among different groups of mice (*n* = 10). (D) Representative vaginal smears. (E) Comparison of the duration of each estrous cycle stage among different groups of mice (*n* = 10). (F–N) qRT‐PCR analysis of *MFN2, BCL2L13, BAX, BCL2, FSHR, LHCGR, CYP11A1, IL6*, and *CXCL1* mRNA expression (*n* = 6). (O–W)Western blot detection of MFN2, BCL2L13, BAX, BCL2, FSHR, LHCGR, CYP11A1, p16 and p21 protein expression (*n* = 3). Quantified protein expression was normalized to β‐actin and expressed relative to the Control group (set as 1.0). Statistical significance was determined using an unpaired two‐tailed Student's *t*‐test or Mann–Whitney *U* test for comparisons between two groups, depending on data normality. ^ns^ *p* > 0.05, *p < 0.05, **p < 0.01, ***p < 0.001.

## Data Availability

The RNA‐seq data generated in this study have been deposited in the Genome Sequence Archive (GSA) of the National Genomics Data Center, China National Center for Bioinformation (CNCB), under accession numbers PRJCA049115 and PRJCA060609. All data supporting the findings of this study are included in the published article and its Supplementary Information files. Additional datasets are available from the corresponding author upon reasonable request. The code used in this study has been deposited in GitHub (https://github.com/zoezoe‐99/jwh).
