# Supplementary material for: Pyroptotic Macrophage Extracellular Vesicles Drive miR‐103a‐3p Mediated Mitochondrial Dysfunction in Granulosa Cells in Diminished Ovarian Reserve
Source: Aging Cell. 2026 Nov 5;25(11):e70806. doi: 10.1111/acel.70806 (PMC13740007; doi:10.1111/acel.70806)
Supplement: Supplementary file 1 [file ACEL-25-e70806-s002.docx]

**Figure S1 Single-cell analysis reveals enhanced pyroptosis of macrophages in aged ovaries and identifies pyroptotic macrophage.**

(A-D) Expression levels of *PYCARD*, *CASP1*, *GSDMD*, and *IL-1B* in macrophages across groups derived from the single-cell RNA-seq dataset GSE267315. (E-F) Measurement of Caspase-1 p20 and GSDMD-N protein expression (n = 3). Quantified protein expression was normalized to β-actin and expressed relative to the M0 group (set as 1.0). Data are presented as mean ± SD. Statistical significance was determined using an unpaired two-tailed Student’s t-test. ^ns^*p*>0.05,^*^*p* < 0.05, ^**^*p* < 0.01,^***^*p* < 0.001.

**Figure S2 Pyr-EVs induce mitochondrial dysfunction and apoptosis in KGN cells.**

(A)qRT-PCR analysis of miR-103a-3p levels (n = 6). (B-F) Measurement of ROS, TMRE, ATP, MDA, and GSH levels (n = 5). (G) qRT-PCR analysis of miR-103a-3p levels (n = 6). (H-I) Flow cytometry (Annexin V-FITC/PI double staining) to assess apoptosis (n = 3). (J-K) Flow cytometry (DHE staining) to detect intracellular ROS levels (n = 3). (L-M) TMRE staining and flow cytometry for mitochondrial membrane potential quantification (n = 3). (N-P) Measurement of ATP, MDA, and GSH levels (n = 5). (Q-R) SA-β-gal staining to assess cellular senescence (n = 9). Data are presented as mean ± SD. Statistical significance was determined using an unpaired two-tailed Student’s t-test for comparisons between two groups and one-way ANOVA followed by Tukey’s multiple comparisons test for comparisons among four groups. ^ns^*p*>0.05,^*^*p* < 0.05, ^**^*p* < 0.01,^***^*p* < 0.001.

**Figure S3 miR-103a-3p levels in human follicular fluid EVs correlate with ovarian reserve and ART outcomes.**

(A-H) Western blot analysis of BCL2, BAX, FSHR, LHCGR, CYP11A1, p21 and p16 protein expression in follicular fluid granulosa cells from DOR and NOR patients (n = 3). Quantified protein expression was normalized to β-actin and expressed relative to the NOR group (set as 1.0). Statistical significance was determined using the Mann-Whitney U test for comparisons between two groups. (I) Multiple linear regression analyses between FF-EVs miR-103a-3p levels and Gn dose, age, group. (J-N) Correlation of miR-103a-3p expression levels in FF-EVs with trigger-day E_2_, retrieved oocytes, fertilized oocytes, normal fertilized oocytes, embryos, bFSH, bLH, bE_2_, and trigger-day LH, trigger-day progesterone levels. ^ns^*p*>0.05,^*^*p* < 0.05, ^**^*p* < 0.01,^***^*p* < 0.001.

**Figure S4 miR-103a-3p induces mitochondrial dysfunction in granulosa cells by targeting and inhibiting MFN2 and BCL2L13**

(A) qRT-PCR analysis of miR-103a-3p levels (n = 6). (B) Measurement of MDA levels (n = 5). (C-G) qRT-PCR analysis of *BCL2, BAX, FSHR, LHCGR,* and *CYP11A1* mRNA expression (n = 5). (H-N) Western blot detection of BCL2, BAX, FSHR, LHCGR,CYP11A1, p16 and p21 protein expression (n = 3). Quantified protein expression was normalized to β-actin and expressed relative to the miRNA NC group (set as 1.0). Statistical significance was determined using an unpaired two-tailed Student’s t-test or Mann-Whitney U test for comparisons between two groups, depending on data normality.^ns^*p*>0.05,^*^*p* < 0.05, ^**^*p* < 0.01,^***^*p* < 0.001.

**Figure S5 Overexpression of MFN2 and BCL2L13 reverses miR-103a-3p-induced impairment of granulosa cell dysfunction under oxidative stress conditions**

(A) KGN cells were treated with varying concentrations of 3-NP for different durations, and cell viability was assessed using the CCK-8 assay (n = 3). (B) KGN cells were exposed to different concentrations of 3-NP for 24 hours, and cell viability was measured using the CCK-8 assay (n = 3). (C-D) Flow cytometry (Annexin V-FITC/PI double staining) to assess apoptosis (n = 3).(E-G) Measurement of ATP, GSH, and MDA levels (n = 5).(H-M) Flow cytometry (DHE staining, MitoSOX staining, TMRE staining) to detect intracellular ROS, mitochondrial ROS, mitochondrial membrane potential quantification (n = 3). (N-O) TEM analysis of mitochondrial morphology; mitochondrial aspect ratio (length/width) (n = 30) and mitochondrial number per cell (n = 6). (P) qRT-PCR analysis of miR-103a-3p levels (n = 6). (Q-S) Measurement of ATP,GSH, and MDA levels (n = 5). (T-Z) qRT-PCR analysis of *MFN2, BCL2L13, BCL2, BAX, FSHR, LHCGR,* and *CYP11A1* mRNA expression (n = 5). (AA-AH) Western blot detection of MFN2, BCL2L13, BAX, BCL2, FSHR, LHCGR, p16 and p21 protein expression (n = 3). Quantified protein expression was normalized to β-actin and expressed relative to the OS+miRNA NC group (set as 1.0). Data are presented as mean ± SD. Statistical significance was analyzed using one-way ANOVA followed by Tukey’s multiple-comparison test, as appropriate. ^ns^*p*>0.05,^*^*p* < 0.05, ^**^*p* < 0.01,^***^*p* < 0.001.

**Figure S6 Inhibition of miR-103a-3p ameliorates oxidative stress-induced ovarian injury in mice**

(A) Fluorescence co-localization analysis confirmed the distribution of AAV in mouse ovarian cells 4 weeks after intraperitoneal injection. (B)Western blot detection of Luciferase protein expression (n = 3). Since Luciferase is inserted exogenously via the AAV vector, it can serve as a reporter for the inhibitory effect of miR-103a-3p. (C) Comparison of body weight among different groups of mice (n = 10). (D) Representative vaginal smears. (E) Comparison of the duration of each estrous cycle stage among different groups of mice (n=10). (F-N) qRT-PCR analysis of *MFN2, BCL2L13, BAX, BCL2, FSHR, LHCGR,* *CYP11A1, IL6,* and *CXCL1* mRNA expression (n = 6). (O-W)Western blot detection of MFN2, BCL2L13, BAX, BCL2, FSHR, LHCGR, CYP11A1, p16 and p21 protein expression (n = 3).Quantified protein expression was normalized to β-actin and expressed relative to the Control group (set as 1.0). Statistical significance was determined using an unpaired two-tailed Student’s t-test or Mann-Whitney U test for comparisons between two groups, depending on data normality. ^ns^*p*>0.05,^*^*p* < 0.05, ^**^*p* < 0.01,^***^*p* < 0.001.
